# Efficacy of eHealth Interventions for Hemodialysis Patients: Systematic Review and Meta-Analysis

**DOI:** 10.2196/67246

**Published:** 2025-03-26

**Authors:** Xu-Hua Zhou, Hui Chen, Weiwei Yang, Li Wang, Lin Chen, Ying Zhu, Yingjun Zhang, Mei Shi, Qin Zhang

**Affiliations:** 1 Hemodialysis Center Department of Nephrology West China Hospital, Sichuan University Chengdu China; 2 West China School of Nursing Sichuan University Chengdu China; 3 Department of Gastroenterology The First Affiliated Hospital of Chengdu Medical College Chengdu China

**Keywords:** hemodialysis, eHealth interventions, quality of life, treatment adherence, anxiety, depression, meta-analysis, kidney, systematic review, kidney diseases, kidney function, chronic diseases

## Abstract

**Background:**

Within hemodialysis patient populations, eHealth interventions have been considered as an alternative and complementary option to routine care services. However, the efficacy of eHealth interventions for hemodialysis patients remains poorly understood owing to a lack of rigorous quantitative evidence synthesis.

**Objective:**

This meta-analysis aimed to evaluate the efficacy of eHealth interventions in improving quality of life, treatment adherence, and psychological outcomes (anxiety and depression) among hemodialysis patients. In addition, the study sought to identify specific intervention components and methodological quality associated with enhanced quality of life and health outcomes in this population.

**Methods:**

A comprehensive search was performed across PubMed, Web of Science, Embase, CINAHL, Cochrane Library, PsycINFO, China National Knowledge Infrastructure, WanFang, China Science and Technology Journal Database, and China BioMedical Literature Database databases from their inception to September 7, 2024. Randomized controlled trials on eHealth interventions for hemodialysis patients published in English or Chinese were included. Critical appraisal was carried out independently by 2 reviewers to assess the bias risk of the studies included. Quantitative synthesis of the outcomes of interest was conducted using a random-effects model. The quality of evidence for the outcomes was evaluated following the Grading of Recommendations, Assessment, Development, and Evaluation approach.

**Results:**

A total of 17 randomized controlled trials involving 1728 participants were included in this meta-analysis out of 5741 articles identified in the initial database search and additional search references. In the 17 studies, 8 kinds of eHealth intervention delivery formats were used, including text messages, telephone sessions, video, network platforms, social media, computers, websites, and mobile apps. The majority of research studies used a single form of eHealth intervention, and 7 studies adopted a combined approach of 2 or more eHealth technologies. The duration of eHealth interventions demonstrated substantial variability across studies, spanning from 4 weeks to 12 months, of which 3 months was the most common. A total of 14 (82%) studies were considered to have “some concern” about selection bias. In addition, 15 (88%) trials were classified as having a “high risk” of performance and detection bias, and all trials were judged to be at “low risk” of attrition and reporting bias. The pooled results revealed a significant difference between the eHealth interventions and control groups on quality of life (standardized mean difference [SMD]=0.87, 95 % CI 0.38 to 1.37, low certainty evidence), treatment adherence (SMD=1.11, 95 % CI 0.30 to 1.91, moderate certainty evidence), anxiety (SMD=–2.11, 95 % CI –3.25 to –0.97, moderate certainty evidence), and depression (SMD=–2.46, 95 % CI –3.68 to –1.25, moderate certainty evidence).

**Conclusions:**

eHealth interventions could be a beneficial approach for improving quality of life and treatment adherence and reducing anxiety and depression among hemodialysis patients. However, future high-quality randomized controlled trials are essential to draw more reliable conclusions.

**Trial Registration:**

PROSPERO CRD42024589799; https://www.crd.york.ac.uk/PROSPERO/view/CRD42024589799

## Introduction

End-stage renal disease (ESRD), which arises as a consequence of the progression of chronic kidney disease (CKD), is a condition that affects millions globally and is recognized as one of the most widespread and significant chronic diseases [1-3]. Hemodialysis (HD), the primary modality of renal replacement therapy for advanced kidney failures, plays a crucial role in mitigating the otherwise inevitable progressive decline in kidney function [4]. As of 2020, it is estimated that 88.07% of patients with ESRD in the United States were undergoing HD, compared with 46% to 98% of patients in Europe [5,6]. HD functions as a partial replacement for kidney activity and plays a critical role in maintaining water-electrolyte homeostasis and, as a result, significantly contributes to the prolongation of patient survival [7,8]. However, while undergoing long-term HD treatment, patients are often accompanied by adverse effects such as anxiety, depression, and fatigue, which significantly impair their compliance with treatment and quality of life [9,10]. Furthermore, the financial burden on HD patients, their families, and society is typically substantial due to the prohibitive cost of dialysis and loss of productivity [11]. Therefore, effective, accessible, and cost-effective interventions aimed at alleviating the negative emotions experienced by HD patients, while improving their adherence and quality of life, are urgently sought.

With the continual refinement of dialysis technology, the life expectancy of HD patients has increased markedly [12]. As a result, their focus has shifted from merely extending survival to enhancing quality of life [13]. To achieve this, HD patients must comply with a strict and well-structured treatment protocol that comprises regular medication intake, diet management, fluid control, and physical activity [14]. However, given the poor accessibility of medical resources and inadequate health literacy, they frequently find it difficult to obtain appropriate self-management support, including health guidance and supervision, psychological counseling, and scheduled follow-up visits [15,16]. These barriers tend to precipitate adverse emotions, such as anxiety and depression, which diminish treatment adherence and ultimately impede the enhancement of the quality of life [17,18]. Furthermore, HD patients primarily receive care services in outpatient clinics and spend the remainder at home or in the community. In contrast, in low- and middle-income countries, underdeveloped community care networks further limit access to timely care and health monitoring, and the effectiveness of their treatments for this condition is usually significantly compromised [19,20].

Benefiting from the rapid development of electronic information technology, eHealth interventions have been recognized as a powerful approach for disease prevention, health behavior maintenance, and improved health outcomes owing to their accessibility and cost-effectiveness [21,22]. In the field of health care, eHealth interventions mainly deliver continuous and personalized services to patients, primarily through digital platforms including the internet, mobile apps, websites, and text messaging [23,24]. Multiple studies have revealed the substantial potential of eHealth interventions, not only in enhancing the quality of life but also in enhancing self-efficacy and significantly reducing anxiety and depression in patients with chronic diseases, thereby underscoring their effectiveness as a multifaceted therapeutic approach capable of addressing both physical and psychological dimensions of patient health [25,26]. However, while a growing body of original research has evaluated the effects of eHealth on quality of life, treatment adherence, anxiety, and depression in HD patients, substantial variability remains in terms of intervention formats, duration, control conditions, and outcome measures [20,27,28]. For instance, a Thailand-based study demonstrated that a tele-home health care model (including video visits, telephone counseling, web-based education, and monitoring) significantly improved the quality of life of patients with HD at 3 months [27]. Similarly, another research conducted in Iran reported that a 1-month period of nurse-delivered tele-nursing significantly reduced the levels of anxiety and depression in HD patients [29]. However, a recent study evaluating the difference between an internet-based self-help intervention and usual care in improving anxiety, depression, and quality of life among HD patients revealed no significant difference in the clinical efficacy of both interventions [28]. Variations in these results may stem from a variety of factors such as different cultural backgrounds, patient characteristics, intervention format, and duration of the intervention. In other words, the mechanisms through which eHealth interventions improve health outcomes in HD patients remain unclear. In addition, the complexity of eHealth interventions also poses serious challenges for health care providers in their implementation [30]. Last but not least, most of the previous research on the application of eHealth in HD patients was conducted with small samples, feasibility trials, or qualitative methods, making it difficult to draw reliable conclusions. To our knowledge, no meta-analyses have been conducted to date that comprehensively investigate the efficacy of eHealth interventions specifically targeting HD patients, highlighting a significant gap in the literature and underscoring the need for comprehensive evidence synthesis in this area. Therefore, a meta-analysis is warranted to clarify the effectiveness of eHealth interventions in HD patients and to identify the optimal form and duration of these interventions for standardized clinical applications in the future.

## Methods

### Design

This meta-analysis was reported in accordance with the Preferred Reporting Items for Systematic Reviews and Meta-Analyses (PRISMA) statement (Multimedia Appendix 1) [31]. The study protocol was registered with PROSPERO (CRD42024589799).

### Literature Search

A literature search was conducted in PubMed, Web of Science, Embase, CINAHL, Cochrane Library, PsycINFO, China National Knowledge Infrastructure (CNKI), WanFang, VIP, and CBM from the inception to September 7, 2024. We used the following search terms: dialysis, hemodialysis, hemodialysis, maintenance hemodialysis, eHealth, website, internet, text messaging, email, digital health, telephone, smartphone, phone, mobile phone, mobile device, mobile health, mHealth, app, application, video, computer, RCT, and randomized controlled trial. The search strategy was designed using Boolean operators to combine relevant terms. For example, in PubMed, the following search string was used: (“dialysis” OR “hemodialysis” OR “haemodialysis” OR “maintenance hemodialysis” OR “maintenance haemodialysis” OR “MHD”) AND (“telemedicine” OR “internet-based intervention” OR “telerehabilitation” OR “web” OR “website” OR “internet” OR “text messaging” OR “email” OR “digital health” OR “eHealth” OR “e-health” OR “telephone” OR “smartphone” OR “phone” OR “mobile phone” OR “mobile device” OR “technology” OR “mobile health” OR “mHealth” OR “m-health” OR “online” OR “app” OR “application” OR “video” OR “computer”) AND (“RCT” OR “randomized clinical trial” OR “randomized controlled trial” OR “randomized trial” OR “randomised controlled trial” OR “randomised trial”). In addition, we complemented the search with a list of references that were incorporated into the literature or relevant definitive reviews. The specific details of all search strategies are contained in Multimedia Appendix 2.

### Inclusion and Exclusion Criteria

The eligibility criteria were determined based on the PICOS (Population, Intervention, Comparison, Outcomes, and Study design) acronym (Textbox 1).

**Eligibility criteria.**

**Inclusion criteria:**

- Population: adult patients (aged 18 years or older) received hemodialysis treatment.
- Intervention: the interventions were administered through a variety of eHealth technologies, including websites, the internet, social media platforms, telephone communications, video consultations, and other digital health tools.
- Comparison: the control groups were assigned to usual care (routine health education and counseling, periodic follow-up, psychological care, and standard physical examinations) without placebo and eHealth technology.
- Outcomes: the study results were focused on the outcomes of quality of life, treatment adherence, and anxiety or depression.
- Study design: adhered to a randomized controlled trial format. In addition, we only included studies published in English or Chinese.

**Exclusion criteria:**

- Duplicate publications.
- Conference abstracts, study protocols, letters, case reports, and reviews.
- Without sufficient data for meta-analysis.

### Study Selection and Data Extraction

All citations were imported into Endnote X20 for data management. Following the import of all references, duplicate entries were removed, after which 2 reviewers independently screened the remaining records by reading the title, abstract, and full text using the predefined inclusion criteria. To ensure methodological rigor in data extraction, 2 independent reviewers (XHZ and HC) retrieved data and recorded them in a structured spreadsheet, which was subsequently cross-verified. From each study, the following data were meticulously extracted: author, publication year, country, age, sample size, details of the experimental and control conditions, duration of the intervention, and measures. In cases where discrepancies arose between the 2 reviewers, a third reviewer (WWY) was consulted to achieve consensus.

### Risk of Bias Assessment

Two reviewers (XHZ and HC) independently assessed the methodological quality of the included studies using the Cochrane Risk of Bias Tool, and any disagreements between them were clarified by discussion with a third researcher (WWY). In cases where consensus could not be reached, the final decision was made by arbitration, with the third reviewer casting the deciding vote. The tool comprises 7 components, including random sequence generation, allocation concealment, blinding of participants and personnel, blinding of outcome assessment, missing outcome data, selective reporting, and other biases [32]. Each domain within the assessment was categorized as presenting either a “low risk,” “unclear risk,” or “high risk” of bias, and for the overall risk of bias evaluation, a study was deemed to carry a “low risk of bias” only if all individual items within the assessment were consistently identified as representing low risk. A study was rated as having “some concerns” when it raised concerns in at least 1 area. It was deemed to have a “high risk of bias” if at least 1 domain exhibited a high risk or if multiple domains indicated concerns.

### Quality of Evidence Assessment

Two researchers separately appraised the quality of evidence for outcomes such as quality of life, treatment adherence, anxiety, and depression, which were evaluated using the Grading of Recommendations, Assessment, Development, and Evaluation (GRADE) framework. Discussion with a third researcher was conducted to clarify disagreements. The framework categorized evidence into 4 levels, “high,” “moderate,” “low,” and “very low” based on limitations in the dimensions of risk of bias, inconsistency, directness, imprecision, and publication bias [33]. In addition, if the effect size was substantial or the dose-response ratio strong, the evidence grade for the outcome could be upgraded by 1 level.

### Statistical Analysis

This study identified quality of life and treatment adherence as primary outcomes and anxiety and depression as secondary outcomes. The meta-analysis and heterogeneity test were conducted with RevMan (version 5.3; Cochrane) and Stata (version 17.0; StataCorp). As the trials measured the outcomes of interest in various tools, the standardized mean difference (SMD) with 95% CI was used to estimate the pooled intervention effect [34]. The SMD magnitudes were interpreted as small (<.5), moderate (.5-.8), and large (>.8) [35]. The *I*^2^ statistic and *P* values were used to evaluate heterogeneity. A fixed-effect model would be applied to collapsed data if *I*^2^ ≤50% and *P*>.1, otherwise a random-effect model would be performed to provide more reliable estimates [34]. In addition, we conducted a subgroup analysis stratified the duration and format of interventions to identify potential contributors to heterogeneity [36]. Given that over 10 studies reported quality of life as an outcome, publication bias was evaluated both visually using a funnel plot and quantitatively through the Egger linear regression method. The trim and fill analysis was performed to adjust for any publication bias and to estimate its effect on effect sizes. Finally, a sensitivity analysis was undertaken to assess the stability of the pooled results by applying a leave-one-out approach.

## Results

### Study Selection

**Figure 1:**
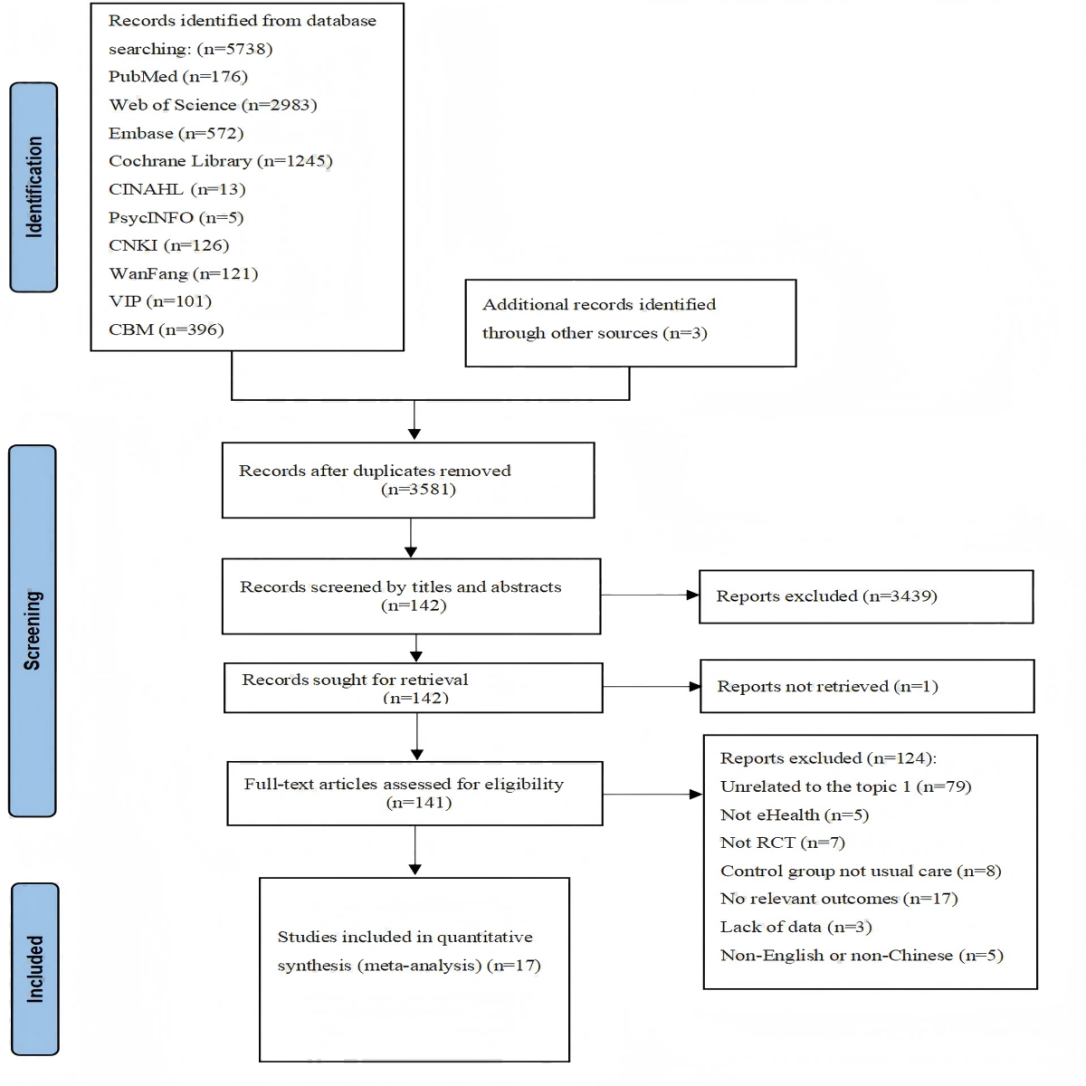
PRISMA flowchart of study selection and literature screening process. RCT: randomized controlled trial.

The initial database search resulted in the retrieval of 5738 articles, with an additional 3 studies identified through a manual review of reference lists. After removing duplicates, 3581 articles were reviewed based on their titles and abstracts. As a result, 142 articles met the initial validation criteria. Subsequently, following a full-text examination, 17 papers were included in this review. The study selection and literature screening process are detailed in Figure 1.

### Characteristics of the Included Studies

A total of 1728 HD patients were recruited in 17 trials [10,17,20,27,28,37-48], of which 879 were assigned to the experimental group based on eHealth technologies, while 849 were in the control group and received usual care. Of the 17 randomized controlled trials, 2 were clustered trials, 1 was a three-arm trial, and 2 were feasibility trials. The studies were conducted in China (n=8), Iran (n=4), Australia (n=1), Turkey (n=1), Oman (n=1), the Netherlands (n=1), Thailand (n=1), with the publication years spanning between 2015 and 2024. The mean ages of all participants ranged from 27 (SD 11.5) to 69.13 (SD 11.82) years old. Nevertheless, three studies failed to provide the age of the participants [20,37,38]. Multimedia Appendix 3 details the characteristics of the included studies.

### Main Features of Interventions

In the 17 studies, 8 kinds of eHealth intervention delivery formats were used, including text messages, telephone sessions, video, network platforms, social media, computers, websites, and mobile apps. The majority of research used a single form of eHealth intervention, and 7 studies adopted a combined approach of 2 or more eHealth technologies [17,20,28,37-40]. The duration of eHealth interventions demonstrated substantial variability across studies, spanning from 4 weeks to 12 months, of which 3 months was the most common.

### Main Features of Controls

All HD patients in the control group were assigned to receive usual care during the intervention, including health assessment, advice and counseling, laboratory and physical examinations, and follow-up care. Nevertheless, there were large gaps between studies in terms of descriptions of the components of usual care, with 6 of the studies [10,20,27,39,41,42] not detailing the elements of the intervention in the control group.

### Outcome Measures

In total, 7 validated scales were used to measure the quality of life among HD patients: the EuroQol 5-Dimension (EQ-5D) [41], the 36-Item Short Form Health Survey (SF-36) [10,37,43-45], the 12-Item Short Form Health Survey (SF-12) [28], the Kidney Disease Quality of Life–Short Form (KDQOL–SF) [39,42], the 9‑item Thai Health Status Assessment Instrument [29], the World Health Organization Quality Of Life-BREF (WHOQOL-BREF) [40], and World Health Organization Quality Of Life-SF (WHOQOL-SF) [20].

Five studies that reported on treatment adherence in HD patients applied 2 various instruments to measure this indicator, including the End-Stage Renal Disease Adherence Questionnaire (ESRD-AQ) [17,20] and the Treatment adherence scale for maintenance hemodialysis patients with end-stage renal disease [44,47].

Three scales were administered to determine HD participants’ anxiety: the Depression Anxiety and Stress Scale (DASS) [27], the Self-Rating Anxiety Scale (SAS) [45-47], and the Beck Anxiety Inventory (BAI) [28,48].

Four solid instruments were used to assess the level of depression among HD patients: DASS [27], the Self-rating Depression Scale (SDS) [45-47], the Beck Depression Inventory-II (BDI-II) [28,48], and the Beck Depression Inventory-Short Form (BDI-SF) [20].

### Risk of Bias

**Figure 2:**
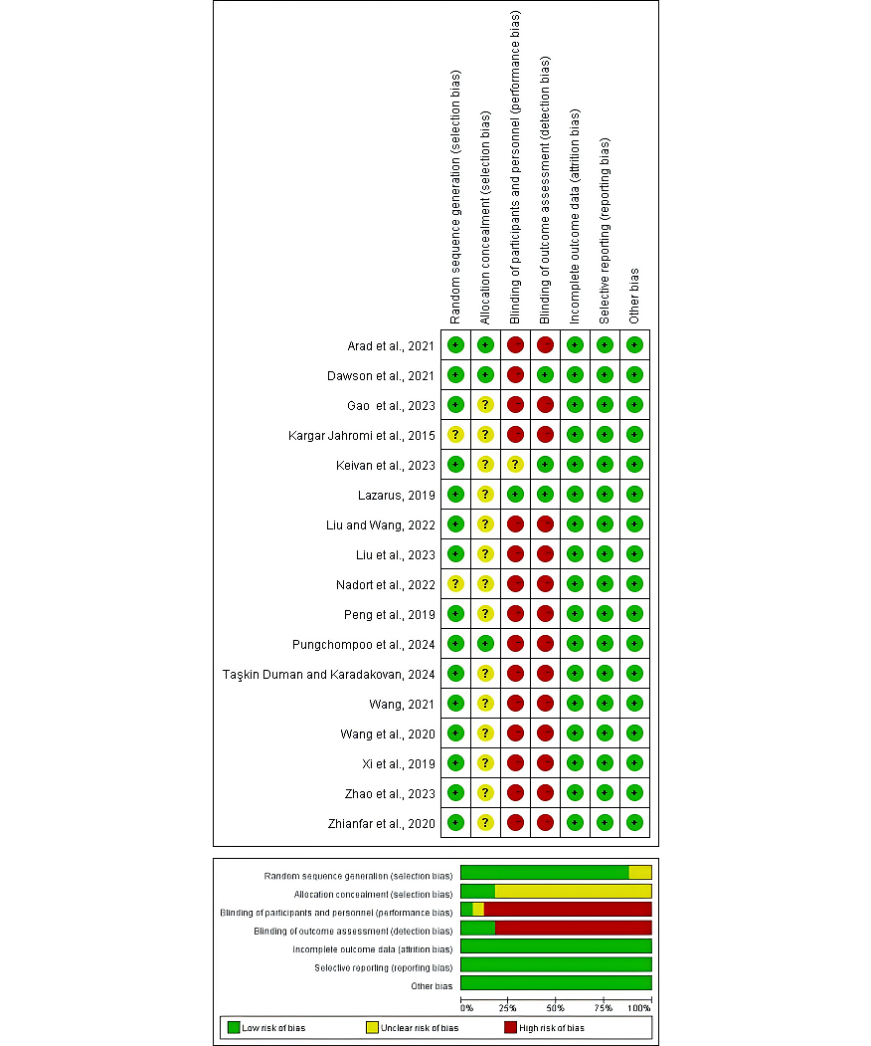
Risk of bias assessment summary of included studies.

A total of 14 studies were considered to have “some concern” about selection bias due to not reporting the specific methods used for random sequence generation or the details of allocation concealment [10,20,27,28,37,39,40,42-48]. In addition, 15 studies were considered to be at “high risk” of performance and detection bias as they did not apply blinding to participants, personnel, or outcome assessors [10,17,20,27-29,37,40,41,43-48]. Ultimately, all trials were judged as having a low risk of attrition and reporting bias because of their low rate of missing outcome data and explicit reasons for missing data, as well as the reported results were consistent with those described in the published study protocols or in the methods sections of the articles. A summary of the risk of bias assessment for the included studies is detailed in Figure 2.

### Main Results of Meta-Analysis

#### Quality of Life

Twelve trials investigated the effect of eHealth interventions on the quality of life of HD patients [10,20,28,37-45]. The pooled result demonstrated a statistically significant efficacy of eHealth interventions in enhancing quality of life, with a substantial effect size (SMD 0.87, 95% CI 0.38-1.37; Multimedia Appendix 4). The sensitivity analysis indicated that no single trial was sufficient to alter the overall result. The sensitivity analysis confirmed the robustness of the meta-analysis findings, as the exclusion of any individual study did not alter the overall results (Figure S2a in Multimedia Appendix 5).

In the analysis categorized by intervention duration, eHealth interventions of short duration (≤3 months) were effective in improving quality of life among HD patients, with a substantial reduction in heterogeneity (SMD 0.5, 95% CI 0.28-0.73; *I*^2^=61%; Multimedia Appendix 4). However, it showed a significantly higher positive impact on quality of life than long-term (>3 months) eHealth interventions (SMD 2.25, 95% CI 0.29-4.21; *I*^2^=98%; Multimedia Appendix 4). Subgroup analysis of the different intervention formats revealed that a single eHealth intervention (SMD 1.5, 95% CI 0.45-2.55; *I*^2^=97%; Multimedia Appendix 4) had a greater positive impact on quality of life than a combination of eHealth interventions.

#### Treatment Adherence

Five trials evaluated the impact of eHealth interventions on adherence among HD patients [17,20,44,47,48]. The pooled results demonstrated that eHealth interventions significantly enhanced HD patients’ treatment adherence (SMD 1.11, 95% CI 0.3-1.91; Multimedia Appendix 4). The sensitivity analysis demonstrated the robustness of the results (Figure S2b in Multimedia Appendix 5).

In the analysis classified by intervention duration, the short-term (≤3 months) eHealth interventions were effective in terms of treatment adherence among HD patients (SMD 1.34, 95% CI 0.31-2.37; *I*^2^=95%), whereas long-term (>3 months) eHealth interventions failed to demonstrate a significant impact (SMD 0.25, 95% CI −0.11 to 0.61; Multimedia Appendix 4). When comparing the relative effects between subgroups of different intervention formats in terms of treatment adherence, it was found that neither single (SMD 0.64, 95% CI −0.05 to 1.33; *I*^2^=91%) nor combined eHealth interventions (SMD 1.89, 95% CI −0.69 to 4.48; *I*^2^=97%) had a significant effect on treatment adherence (Multimedia Appendix 4).

#### Anxiety

Six trials provided sufficient data about the impact of eHealth interventions on anxiety among HD patients [27,28,45-48]. The pooled results revealed that eHealth interventions led to a significant reduction in anxiety levels among HD patients (SMD −2.11, 95% CI −3.25 to −0.97; Multimedia Appendix 4). Despite the very high heterogeneity of the combined results, sensitivity analysis confirmed the stability of the result (Figure S2c in Multimedia Appendix 5). When comparing the effects between subgroups of different intervention duration on anxiety of HD patients, short-term (≤3 months) eHealth interventions (SMD −1.95, 95% CI −3.84 to −0.07; *I*^2^=98%) had significantly lower pooled effects than long-term (≤3 months) eHealth interventions (SMD −2.29, 95% CI −3.74 to −0.83; *I*^2^=95%; Multimedia Appendix 4). In addition, the subgroup analyses revealed that single eHealth interventions significantly reduced anxiety in HD patients (SMD −2.54, 95% CI −3.71 to −1.39; *I*²=96%), while mixed eHealth interventions showed no statistically significant impact (SMD 0.08, 95% CI −0.28 to 0.43; Multimedia Appendix 4).

#### Depression

Seven trials reported adequate data on the effect of eHealth interventions on anxiety among HD patients [20,27,28,45-48]. The pooled results showed that eHealth interventions had a statistically significant impact on reducing depression, with a large effect size (SMD −2.46, 95% CI −3.6 to −1.25; Multimedia Appendix 4). The sensitivity analysis revealed that the results remained consistent, with no significant changes after excluding any individual studies (Figure S2d in Multimedia Appendix 3).

When comparing the effects between subgroups of different intervention duration on depression of HD patients, short-term (≤3 months) eHealth interventions (SMD −1.69, 95% CI −3.03 to −0.35; *I*^2^=97%) had significantly lower pooled effects than long-term (≤3 months) eHealth interventions (SMD −3.53, 95% CI −6.50 to −0.57; *I*^2^=98%; Multimedia Appendix 4). Furthermore, a subgroup analysis stratified by intervention formats revealed that a single eHealth intervention was more effective in reducing anxiety in HD patients (SMD −3.40, 95% CI −5.07 to −1.72; *I*^2^=95%; Multimedia Appendix 4).

#### Publication Bias

We used the Egger test and a funnel plot to evaluate potential publication bias in studies related to quality of life. The asymmetry observed in the funnel plot indicated the presence of publication bias among the included studies (Figure S3 in Multimedia Appendix 6). Furthermore, the results of the Egger test indicated a significant bias, with a regression coefficient (beta1) of 13.92 (SE 2.084). The corresponding *z* value was 6.68, and the *P* value was <.001, suggesting that small-study effects are present in the analysis. This result (P<.001) indicates that the observed effect size may be influenced by small sample studies, which could lead to potential publication bias. Thus, the trim-and-fill analysis was performed to counteract the bias. However, there were no missing studies during the analysis and the corrected effect sizes remained unchanged, indicating the stability of the meta-analysis results.

#### Quality of Evidence

Following the guidance in the GRADE standard, the certainty of the evidence for the quality of life, treatment adherence, anxiety, and depression were classified as low, moderate, moderate, and moderate, respectively. The justification for the ratings and other details are listed in Table 1.

**Table 1:** Grading of Recommendations, Assessment, Development, and Evaluation summary of the quality of the evidence for the outcomes.

Outcome	Quality assessment						Number of participants (studies)		Effect size, SMD (95% CI)		Quality of the evidence (GRADE^a^)
	Risk of bias	Inconsistency	Indirectness	Imprecision	Other considerations						
Quality of life	Serious^b^	Serious^c^	Not serious^d^	Not serious^e^	Publication bias strongly suspected^f^ large effect size^g^	1243 (12)		0.87 (0.38 to 1.37)		⊕⊕○○ Low	
Treatment adherence	Serious^b^	Serious^c^	No serious^d^	No serious^e^	Large effect size^g^	508 (5)		1.11 (0.3 to 1.91)		⊕⊕⊕○ Moderate	
Anxiety	Serious^b^	Serious^c^	No serious^d^	No serious^e^	Large effect size^g^	536 (6)		−2.11 (−3.25 to −0.97)		⊕⊕⊕○ Moderate	
Depression	Serious^b^	Serious^c^	No serious^d^	No serious^e^	Large effect size^g^	634 (7)		−2.46 (−3.68 to −1.25)		⊕⊕⊕○ Moderate	

^a^GRADE: Grading of Recommendations, Assessment, Development, and Evaluation.

^b^Most information is from studies rated as high risk of bias.

^c^*I*^2^>50%; heterogeneity could not be explained by the form and duration of intervention.

^d^The various eHealth interventions are compared directly with usual care among cancer patients.

^e^The 95% CI excludes pooled effect sizes that are not clinically significant.

^f^The results of the Egger test and funnel plot revealed the evidence for publication bias.

^g^The pooled effect size >0.8.

## Discussion

### Principal Findings

As far as we know, this study was the first meta-analysis of randomized controlled trials to systematically assess the efficacy of eHealth interventions on quality of life, treatment adherence, anxiety, and depression among HD patients. A total of 17 trials involving 1728 HD patients were included in this meta-analysis. We found varied quality of evidence for the effects of eHealth interventions on quality of life, treatment adherence, anxiety, and depression in people with HD. Specifically, low-quality evidence demonstrated that eHealth interventions have significant clinical effects in enhancing the quality of life among HD patients compared with the control groups. In addition, moderate quality evidence indicated that eHealth interventions can significantly enhance treatment adherence and reduce anxiety and depression in HD patients. However, given the worrying overall methodological quality, more high-quality clinical trials remain essential to draw reliable conclusions in the future. Specifically, the majority of studies included in the meta-analysis had selection, performance, and detection biases, and these potential biases may limit the generalizability of the findings to a wider population, and the lack of blinding may have led to exaggerated estimates of the efficacy of the eHealth interventions. In addition, the subjectivity of the outcome measures combined with unblinded assessments may have affected the results. Furthermore, although the sensitivity analysis results indicated the robustness of the estimated outcomes, significant heterogeneity was still observed that was difficult to explain through subgroup analysis. First, differences in the methodological quality of the included studies, particularly inconsistencies in randomization and blinding, may have contributed to the variability in the results. Second, differences in sample characteristics, such as age, gender, and disease severity, could also be an important source of heterogeneity. Finally, different measurement tools and evaluation standards may lead to the same variable being recorded and assessed in various ways, thereby increasing the inconsistency of the results.

Our findings demonstrated a large effect of eHealth interventions on enhancing the quality of life among HD patients, consistent with a previous meta-analysis evaluating the impact of eHealth intervention on the quality of life of breast cancer patients [49]. Currently, the Internet, telephones, and mobile apps are essential components of people’s daily lives, and they have laid a good foundation for the application of eHealth interventions in HD patients [29]. According to the biopsychosocial model, the individual health status is the consequence of the interaction of biological, psychological, and social factors [50]. The timeliness, rapidity, and accuracy of eHealth interventions enable HD patients to receive continuous care, based on which they can constantly monitor their physiological status and reduce complications [27,28]. Furthermore, through channels such as email and mobile apps, HD patients are able to receive psychological support from family and society as well as psychological counseling from health care professionals, thus enhancing mental health [46]. Finally, over the internet, social media, or apps, HD patients could seek help from health care professionals (health counseling, psychosocial support, and visit coordination), thus improving social adaptation [48]. These positive aspects of improvement in physical, psychological, and social factors ultimately boost the overall health of the patient and achieve an overarching quality of life [37,41]. However, the estimated effect size of eHealth interventions on quality of life exhibited substantial heterogeneity. When the study by Zhao et al [45] was excluded, a significant reduction in the overall effect size was observed, with *I*² decreasing from 93.87% to 79.9%. This suggests that a substantial impact was made on the overall result of this study, which may have been a significant source of the detected heterogeneity. The potential overestimation of the intervention effect in the study of Zhao et al [45] may be attributed to the lack of rigorous randomization and blinding procedures. Therefore, the effectiveness of eHealth interventions on the quality of life of HD patients should be carefully considered. In addition, it is noteworthy that the subgroup analyses indicated that a long-term and single form of eHealth intervention appeared to be more efficacy in enhancing HD patients’ quality of life. This could be because HD patients improve their disease management knowledge and skills over time as the intervention progresses, which helps in effectively preventing and reducing complications. However, if the intervention is overly complex and time-consuming, such as involving multiple eHealth technologies that require extensive eHealth literacy, it may lead to poor patient adherence and potentially increase the overall burden on the patient [49]. Therefore, when implementing eHealth interventions for HD patients, careful consideration should be given to optimizing the duration and format of the intervention to avoid placing unnecessary burdens on patients.

In line with findings on quality of life, our findings demonstrated that eHealth interventions exerted a favorable effect on treatment adherence among HD patients. Treatment adherence among HD patients primarily encompasses dialysis schedule, prescribed session time, medication regimens, and dietary restrictions (namely salt and water intake) [14,47]. In recent years, eHealth interventions have emerged as an effective approach for assessing, monitoring, and managing self-care activities in HD patients [51,52]. The Health Belief Model holds that an individual’s health behaviors depend on his or her perception of the threat of disease and assessment of the benefits of and barriers to behavioral modification [53]. Through the vehicle of eHealth technology (mobile health apps and remote monitoring), HD patients can maintain closer contact with health care professionals, facilitating access to personalized health information and reminders, thus increasing awareness of the severity of the disease and the imperative for treatment [17,20]. In addition, eHealth platforms make it possible to systematically collect health data from HD patients, and with real-time feedback and visualization of data (eg, dialysis outcomes and lab metrics), it is easier for patients to perceive the benefits of their treatment, thus increasing their insights into treatment adherence [48]. In subgroup analyses, short-term eHealth interventions seem to be more effective in enhancing treatment adherence among HD patients. Furthermore, no statistically significant difference was observed in the impact on treatment adherence among HD patients, regardless of whether they received a single form of eHealth intervention or a combination of multiple forms. This underscores the ongoing need to further investigate the optimal timing and format of eHealth interventions to enhance treatment adherence among HD patients in the future.

Due to the severe burden of the disease, HD patients generally exhibit high levels of anxiety [54]. Although routine nursing interventions may temporarily reduce anxiety, their effectiveness commonly declines over time due to a lack of continuity, leading to decreased compliance and affecting patients’ quality of life [55]. This review identified a significant impact of eHealth interventions in alleviating anxiety among HD patients. This finding might be explained through self-efficacy theory. Self-efficacy refers to the individual’s belief in his or her ability to successfully perform the necessary behaviors to achieve a desired outcome [56]. Monitoring, reminding and feedback were the most common eHealth intervention components in the included studies, and these features provide HD patients with the tools and resources to actively manage their disease. When patients perceive tangible gains in their health status, their self-efficacy increases, resulting in a reduction in anxiety associated with disease management [46]. Subgroup analyses indicated that anxiety reductions were more pronounced when patients received eHealth interventions for over 3 months, compared with shorter-term interventions. In addition, a single form of eHealth intervention showed statistically significant effects in reducing anxiety among HD patients, whereas the impact of combined interventions was not significant. However, the effectiveness of eHealth interventions in alleviating anxiety among HD patients requires further validation through more rigorous clinical trials, given the limited number of studies using combined intervention formats, the heterogeneity of interventions, and the moderate quality of the current evidence.

Similar to the pooled results of anxiety, our findings suggested that eHealth interventions had a significant efficacy in decreasing depression among HD patients. Consistent with our findings, a previous study reported that providing psychological counseling services, along with sharing health knowledge and communication skills through a WeChat chatbot, can effectively alleviate depression in HD patients [45]. However, another study found no statistically significant effect of web-based problem-solving therapy on depression levels among HD patients [28]. This inconsistency may be explained by variations in the duration and format of eHealth interventions across different studies. According to the Stress and Coping Theory, equipping patients with effective coping strategies, such as emotional support and problem-solving skills, to help them manage stress could reduce the psychological burden associated with chronic illness [57]. In the included studies, psychological counseling was frequently a crucial element of eHealth intervention, and with the online platform, health care professionals were able to support patients in venting their negative emotions promptly, thereby reducing depression levels. The subgroup analyses revealed that reductions in depressive symptoms were more pronounced when participants received eHealth interventions lasting longer than 3 months or delivered in a single format. The prevalence of depression in HD patients has been estimated at 56.8% [58], which highlights the urgency of targeted interventions. As a chronic mental condition, depression typically requires sustained and consistent interventions to achieve significant improvements [59]. eHealth interventions shorter than 3 months may be insufficient for alleviating depression in HD patients, and combined interventions may present challenges in implementation. Therefore, extending the duration and increasing the frequency of interventions are recommended in future studies to facilitate the demission of depression.

### Limitations

Some limitations should be considered. First, this study only included randomized controlled trials published in English and Chinese, with no incorporation of gray literature such as unpublished studies, conference abstracts, and theses. This exclusion may have contributed to publication bias, particularly in the evaluation of quality of life, as gray literature often includes negative or null results that are less likely to be published in peer-reviewed journals. Consequently, the omission of such studies may have led to an overestimation of the intervention effect. Furthermore, the lack of grey literature may have reduced the diversity of the evidence base, potentially underestimating the heterogeneity among studies and limiting the generalizability of the findings to broader populations or settings. Second, the substantial heterogeneity observed in the pooled outcomes of interest, which resulted from the considerable differences in methodological quality and clinical characteristics of the included patients, could not be clearly explained by subgroup analysis, as no definitive source of heterogeneity was identified. Finally, as the majority of studies included in the meta-analysis were conducted in low- and middle-income countries, the applicability of the findings to high-income countries may be limited.

### Implications

eHealth interventions have attracted growing attention worldwide, with numerous studies highlighting their significant potential in the health care of chronically ill patients [23,56]. This meta-analysis provided the first quantitative synthesis of research findings on the administration of eHealth interventions in HD patients and found that these interventions were effective in improving quality of life and treatment adherence, as well as reducing anxiety and depression levels. These findings emphasize the value of integrating eHealth interventions into clinical nursing practice as a valuable and effective strategy. Despite significant heterogeneity in the included studies, this study consolidated the findings of the impact of eHealth interventions on different health-related outcomes among HD patients and would constitute valuable evidence for health care practitioners and researchers to understand the range of areas where eHealth interventions could make a difference, and the magnitude of the impacts that they would have made. While additional research is required to validate these findings, eHealth interventions continue to show great promise in enhancing quality of life, improving treatment adherence, and alleviating anxiety and depression in HD patients. In addition, we also observed that the duration and format of eHealth interventions had a remarkable effect on their efficacy, prompting the notion that we might have to determine the optimal balance to foster patient health outcomes. Notably, the methodological appraisal revealed a high risk of bias in nearly all included studies, underscoring the urgent need for improvements in study design and intervention delivery. Therefore, when designing and implementing eHealth interventions in the future, it is crucial to incorporate appropriate strategies to enhance patient motivation and adherence, thereby ensuring the effectiveness of the interventions.

### Conclusions

In conclusion, this meta-analysis highlighted that eHealth interventions may serve as an effective strategy for enhancing quality of life, improving treatment adherence, and alleviating anxiety and depression in HD patients. Thus, health care providers should consider eHealth interventions as a pivotal strategy for facilitating HD patient health outcomes. Furthermore, the efficacy of eHealth interventions for HD patients varied significantly depending on the duration and format of the intervention. Therefore, further in-depth research on eHealth intervention strategies for this population is essential to enhance intervention effectiveness and address the growing health care needs of HD patients. Last but not least, it is essential that future high-quality studies be conducted to provide more robust evidence regarding the clinical benefits of eHealth interventions for HD patients.

## Data Availability

The datasets used in this study are publicly accessible, with detailed information provided in the Methods section.

## Appendix

Multimedia Appendix 1 PRISMA checklist.

Multimedia Appendix 2 Search strategy.

Multimedia Appendix 3 Characteristics of the included studies.

Multimedia Appendix 4 Results of meta-analysis: forest plots for the comparison of eHealth interventions against usual care.

Multimedia Appendix 5 Sensitivity analysis.

Multimedia Appendix 6 Publication bias of included studies.

## Abbreviations

BAI: Beck Anxiety Inventory
BDI-II: Beck Depression Inventory-II
BDI-SF: Beck Depression Inventory-Short Form
CKD: chronic kidney disease
DASS: Depression Anxiety and Stress Scale
EQ-5D: EuroQol 5-Dimension
ESRD: end-stage renal disease
ESRD-AQ: End-Stage Renal Disease Adherence Questionnaire
GRADE: Grading of Recommendations, Assessment, Development, and Evaluation
HD: hemodialysis
KDQOL-SF: Kidney Disease Quality of Life–Short Form
PICOS: Population, Intervention, Comparison, Outcomes, and Study design
PRISMA: Preferred Reporting Items for Systematic Reviews and Meta-Analyses
SAS: Self-Rating Anxiety Scale
SDS: Self-rating Depression Scale
SF-12: 12-Item Short Form Health Survey
SF-36: 36-Item Short Form Health Survey
WHOQOL-BREF: World Health Organization Quality of Life-BREF
WHOQOL-SF: World Health Organization Quality of Life-SF
