# Supplementary material for: Efficacy of eHealth Interventions for Hemodialysis Patients: Systematic Review and Meta-Analysis
Source: J Med Internet Res. 2025 Mar 26;27:e67246. doi: 10.2196/67246 (PMC11988279; doi:10.2196/67246)
Supplement: Multimedia Appendix 2 [file jmir_v27i1e67246_app2.docx]

**Multimedia Appendix 2. The search strategies (Date Run: 7/09/2024)**

| **Databases** | **Step** | **Search Strategies** | **Results** |
| --- | --- | --- | --- |
| PubMed | #1 | "dialysis"[Mesh Terms] Sort by: Most Recent | 24,216 |
|  | #2 | "hemodialysis"[Title/Abstract] OR "haemodialysis"[Title/Abstract] OR "maintenance hemodialysis"[Title/Abstract] OR "maintenance haemodialysis"[Title/Abstract] OR "MHD"[Title/Abstract] Sort by: Most Recent | 94,716 |
|  | #3 | "Telemedicine"[MeSH Terms] Sort by: Most Recent | 49,108 |
|  | #4 | "Internet-Based Intervention "[MeSH Terms] Sort by: Most Recent | 1,376 |
|  | #5 | "Telerehabilitation"[MeSH Terms] Sort by: Most Recent | 1,253 |
|  | #6 | "web"[Title/Abstract] OR "website"[Title/Abstract] OR "internet"[Title/Abstract] OR "text messaging"[Title/Abstract] OR "email"[Title/Abstract] OR "digital health"[Title/Abstract] OR "eHealth"[Title/Abstract] OR "e-health"[Title/Abstract] OR "telephone"[Title/Abstract] OR "smartphone"[Title/Abstract] OR "phone"[Title/Abstract] OR "mobile phone"[Title/Abstract] "mobile device"[Title/Abstract] OR "technology"[Title/Abstract] OR "mobile health"[Title/Abstract] OR "mHealth"[Title/Abstract] OR "m-health"[Title/Abstract] OR "online"[Title/Abstract] OR "app"[Title/Abstract] OR "application"[Title/Abstract] OR "video"[Title/Abstract] OR "computer"[Title/Abstract]  Sort by: Most Recent | 2,196,365 |
|  | #7 | "RCT"[Title/Abstract] OR "randomized clinical trial"[Title/Abstract] OR "randomized controlled trial"[Title/Abstract] OR "randomized trial"[Title/Abstract] OR "randomised controlled trial"[Title/Abstract] OR "randomised trial"[Title/Abstract] Sort by: Most Recent | 277,070 |
|  | #8 | #1 OR #2 Sort by: Most Recent | 118,642 |
|  | #9 | #3 OR #4 OR #5 OR #6 Sort by: Most Recent | 2,223,924 |
|  | #10 | #7 AND #8 AND #9 Sort by: Most Recent | 176 |
| Web of Science | #1 | **Ts=(dialysis) OR TS=(**hemodialysis) OR TS=(haemodialysis) OR TS=(maintenance hemodialysis) OR TS=(maintenance haemodialysis) OR TS=(MHD) | 380,471 |
|  | #2 | **Ts=(web) OR** TS=(website) OR **TS=(internet**) OR TS=(text messaging) OR TS=(email) OR TS=(digital health) OR TS=(eHealth) OR TS=(e-health) OR **Ts=(**telephone**) OR TS=(**smartphone) OR TS=(phone) OR TS=(mobile phone) OR TS=(mobile device) OR TS=(technology) OR **Ts=(**mobile health**) OR TS=(**mHealth) OR TS=(m-health) OR TS=(online) OR TS=(app) OR TS=(application) OR TS=(video) OR TS=(computer) | 14,609,630 |
|  | #3 | TS=(RCT) OR **TS=(**randomized clinical trial**) OR TS=(**randomized controlled trial) OR TS=(randomized trial) OR TS=(randomised controlled trial) OR TS=(randomised trial) | 1,112,490 |
|  | #4 | #1 AND #2 AND #3 | 2,983 |
| EMBASE | **#1** | **'dialysis'/exp** | 250,483 |
|  | **#2** | **'hemodialysis'/exp** | 149,935 |
|  | **#3** | **'haemodialysis'/exp** | 149,935 |
|  | **#4** | '**dialysis':ti,ab,kw OR 'hemodialysis':ti,ab,kw OR 'haemodialysis':ti,ab,kw OR 'maintenance hemodialysis':ti,ab,kw OR 'maintenance haemodialysis':ti,ab,kw OR 'MHD' :ti,ab,kw** | 279,158 |
|  | #5 | **'t**elemedicine**'/exp** | 78,921 |
|  | #6 | **'t**elerehabilitation**'/exp** | 3,254 |
|  | #7 | 'web-based intervention'/exp | 3,557 |
|  | #8 | 'web**':ti,ab,kw OR 'website':ti,ab,kw OR 'internet':ti,ab,kw OR** 'text messaging**':ti,ab,kw OR 'email':ti,ab,kw OR '**digital health**':ti,ab,kw OR** 'ehealth**':ti,ab,kw OR '**e-health**':ti,ab,kw OR '**telephone**':ti,ab,kw OR** 'smartphone**':ti,ab,kw OR 'phone':ti,ab,kw OR '**mobile phone**':ti,ab,kw OR '**mobile cdevice**':ti,ab,kw OR '**technology**' :ti,ab,kw OR** 'mobile health**':ti,ab,kw OR '**mhealth**':ti,ab,kw OR '**m-health**':ti,ab,kw OR** 'online**':ti,ab,kw OR 'app':ti,ab,kw OR 'application':ti,ab,kw OR** 'video**':ti,ab,kw OR 'computer':ti,ab,kw** | 3,173,092 |
|  | #9 | 'RCT**':ti,ab,kw OR '**randomized clinical trial**':ti,ab,kw OR '**randomized controlled trial**':ti,ab,kw OR '**randomized trial**':ti,ab,kw OR '**randomised controlled trial**':ti,ab,kw OR '**randomised trial**':ti,ab,kw** | 379,011 |
|  | #10 | #1 OR #2 OR #3 OR #4 | 341,796 |
|  | #11 | #5 OR #6 OR #7 OR #8 | 3,211,214 |
|  | #12 | #9 AND #10 AND #11 | 572 |
| Cochrane Library | #1 | MeSH descriptor: [Dialysis] explode all trees | 21,246 |
|  | #2 | (dialysis):ti,ab,kw OR (hemodialysis):ti,ab,kw OR (haemodialysis):ti,ab,kw OR (maintenance hemodialysis):ti,ab,kw OR (maintenance haemodialysis):ti,ab,kw OR (MHD):ti,ab,kw | 23,657 |
|  | #3 | MeSH descriptor: [Telemedicine] explode all trees | 7,169 |
|  | #4 | MeSH descriptor: [Telerehabilitation] explode all trees | 1,726 |
|  | #5 | (web)**:ti,ab,kw OR (website):ti,ab,kw OR (internet):ti,ab,kw OR (**text messaging)**:ti,ab,kw OR (email):ti,ab,kw OR (**digital health)**:ti,ab,kw OR (**ehealth)**:ti,ab,kw OR (**e-health)**:ti,ab,kw OR (**telephone)**:ti,ab,kw OR (**smartphone)**:ti,ab,kw OR (phone):ti,ab,kw OR (**mobile phone)**:ti,ab,kw OR (**mobile device)**:ti,ab,kw OR (**technology)**:ti,ab,kw OR (**mobile health)**:ti,ab,kw OR (**mhealth)**:ti,ab,kw OR (**m-health)**:ti,ab,kw OR (**online)**:ti,ab,kw OR (app):ti,ab,kw OR (application):ti,ab,kw OR (**video)**:ti,ab,kw OR (computer):ti,ab,kw** | 254,725 |
|  | #6 | (RCT)**:ti,ab,kw OR (**randomized clinical trial)**:ti,ab,kw OR (**randomized controlled trial)**:ti,ab,kw OR (**randomized trial)**:ti,ab,kw OR (**randomised controlled trial)**:ti,ab,kw OR (**randomised trial)**:ti,ab,kw** | 942,763 |
|  | #7 | #1 OR #2 | 26,094 |
|  | #8 | #3 OR #4 OR #5 | 257,307 |
|  | #9 | #6 AND #7 AND #8 | 1,245 |
| CINAHL | S1 | TI dialysis OR TI hemodialysis OR TI haemodialysis OR TI maintenance hemodialysis OR TI maintenance haemodialysis OR TI MHD | 22139 |
|  | S2 | TI web **OR TI website OR TI internet OR TI** text messaging **OR TI email OR TI** digital health **OR TI** ehealth **OR TI** e-health **OR TI** telephone **OR TI** smartphone **OR TI phone OR TI** mobile phone **OR TI** mobile device **OR TI** technology **OR TI** mobile health **OR TI** mhealth **OR TI** m-health **OR TI** online **OR TI app OR TI application OR TI** video **OR TI computer** | 213125 |
|  | S3 | TI RCT **OR TI** randomized clinical trial **OR TI** randomized controlled trial **OR TI** randomized trial **OR TI** randomised controlled trial **OR TI** randomised trial | 82215 |
|  | S4 | S1 AND S2 AND S3 | 13 |
| PsycINFO | S1 | TI dialysis OR TI hemodialysis OR TI haemodialysis OR TI maintenance hemodialysis OR TI maintenance haemodialysis OR TI MHD | 1864 |
|  | S2 | TI web **OR TI website OR TI internet OR TI** text messaging **OR TI email OR TI** digital health **OR TI** ehealth **OR TI** e-health **OR TI** telephone **OR TI** smartphone **OR TI phone OR TI** mobile phone **OR TI** mobile device **OR TI** technology **OR TI** mobile health **OR TI** mhealth **OR TI** m-health **OR TI** online **OR TI app OR TI application OR TI** video **OR TI computer** | 157740 |
|  | S3 | TI RCT **OR TI** randomized clinical trial **OR TI** randomized controlled trial **OR TI** randomized trial **OR TI** randomised controlled trial **OR TI** randomised trial | 31560 |
|  | S4 | S1 AND S2 AND S3 | 5 |
| CNKI |  | (SU="血液透析" + "维持性血液透析" + "血液透析患者") AND (SU="电话" + "短信" + "互联网" + "app" + "视频" + "网站" + "信息平台" + "远程护理" + "远程康复") | 126 |
| WanFang |  | 题名或关键词:(血液透析 OR 维持性血液透析 OR 血液透析患者) and 题名或关键词:(电话 OR 短信 OR 互联网 OR APP OR 视频 OR 网站 OR 信息平台 OR远程护理 OR 远程康复) | 121 |
| VIP |  | (题名或关键词=血液透析 OR 维持性血液透析 OR 血液透析患者) and (题名或关键词=电话 OR 短信 OR 互联网 OR APP OR 视频 OR 网站 OR 信息平台 OR远程护理 OR 远程康复) | 101 |
| CBM |  | ("血液透析"[常用字段:智能] OR "维持性血液透析"[常用字段:智能] OR "血液透析患者"[常用字段:智能] )AND ("电话"[常用字段:智能] OR "短信"[常用字段:智能] OR "互联网"[常用字段:智能] OR "APP"[常用字段:智能] OR "视频"[常用字段:智能] OR "网站"[常用字段:智能] OR "信息平台"[常用字段:智能] OR "远程医疗"[常用字段:智能] ) | 396 |
