# Supplementary material for: Efficacy of eHealth Interventions for Hemodialysis Patients: Systematic Review and Meta-Analysis
Source: J Med Internet Res. 2025 Mar 26;27:e67246. doi: 10.2196/67246 (PMC11988279; doi:10.2196/67246)
Supplement: Multimedia Appendix 3 [file jmir_v27i1e67246_app3.docx]

**Table 1.** Characteristics of the included studies.

| Author(Year) | Country | Age (years), mean (SD) | Sample size | Control group | Experimental group | | | | |
| --- | --- | --- | --- | --- | --- | --- | --- | --- | --- |
|  |  |  |  |  | Brief introduction | Format | Duration | Follow-ups | Measures |
|  |  |  |  |  |  |  |  |  |  |
| Arad et al [17], 2021 | Iran | EG: 27.00 ± 11.50 CG: 30.00 ± 9.50 | EG: 33 CG: 33 | Usual care | A telemedicine-based follow-up program using teleconferencing and text messaging, with components including diet, medication use, and fluid restriction. | Text message + Telephone | 3 months | Baseline, post-intervention, 1-month post-intervention, 3-months post-intervention | 1.Treatment adherence: ESRD-AQ |
| Dawson et al [41], 2021 | Australia | EG: 64.40 ± 13.20 CG: 65.20 ± 14.50 | EG: 87 CG: 43 | Usual care | Coaching on healthy diet and lifestyle to hemodialysis patients through unidirectional text messages. | Text message | 6 months | Baseline, post-intervention, 3-months post-intervention, 6-months post-intervention | 1.QoL: EQ-5D |
| Gao et al [37], 2023 | China | Not reported | EG: 50 CG: 50 | Usual care | Utilising WeChat and website platforms to carry out online hemodialysis-related health education, consultation and follow-up visits | Network platform + Social media | 6 months | Baseline, post-intervention | 1.QoL: SF-36 |
| Kargar Jahromi et al [27], 2015 | Iran | 69.13 ± 11.82 | EG: 30 CG: 30 | Usual care | Script-based telephone follow-up counselling on communication, nutrition, sleep and other health topics. | Telephone | 1 month | Baseline, post-intervention, 1-month post-intervention | 1.Anxiety: DASS 2. Depression: DASS |
| Keivan et al [39], 2023 | Iran | EG: 52.66 ± 9.23 CG: 52.83 ± 8.71 | EG: 30 CG: 30 | Usual care | A self-management program based on the 5A model with face-to-face meetings, telephone follow-ups and SMS coaching. | Text message + Telephone | 3 months | Baseline, post-intervention, 3-months post-intervention | 1.QoL: KDQOL–SF |
| Lazarus [42], 2019 | Oman | 48.77 ± 10.32 | EG: 75 CG: 75 | Usual care | Participants accessed educational sessions on the causes of CKD, haemodialysis treatments and functional exercises via laptops. | Computer | 2 months | Baseline, post-intervention, 1-month post-intervention, 2-months post-intervention | 1.QoL: KDQOL–SF |
| Liu and Wang [40], 2022 | China | EG: 44.86 ± 9.02 CG: 44.93 ± 9.07 | EG: 40 CG: 40 | Usual care | Implementing the 5A model of care using social media for hemodialysis patients, which includes questioning, assessing, advising, assisting and following up, to facilitate lifestyle changes and good self-management behaviours. | Social media + Telephone | 3 months | Baseline, post-intervention | 1.QoL: WHOQOL-BREF |
| Liu et al [47], 2023 | China | EG: 55.26 ± 6.81 CG: 56.00 ± 6.26 | EG: 60 CG: 60 | Usual care | Dissemination of disease knowledge and provision of targeted psychological interventions for participants via a WeChat bot interaction platform. | Social media | 4 months | Baseline, post-intervention | 1. Treatment adherence: Medical Treatment Adherence Scale for the End-stage Renal Disease Patients with Maintenance Hemodialysis 2.Anxiety: SAS 3.Depression: SDS |
| Nadort et al [28], 2022 | Netherlands | EG: 63.00 ± 15.00 CG: 65.00 ± 15.00 | EG: 89 CG: 101 | Usual care | An self-help problem-solving therapy consists of five modules,such as information, examples and assignments. | Website + Computer | 12 weeks | Baseline, post-intervention, 12-weeks post-intervention | 1.QoL: SF-12 2.Anxiety: BAI 3.Depression: BDI-II |
| Peng et al [43], 2019 | China | EG: 57.39 ± 10.32 CG: 57.44 ± 10.30 | EG: 110 CG: 110 | Usual care | Monitoring the health status of hemodialysis patients by utilising a mobile application, with patients regularly uploading health data for personalised care plans to be developed by healthcare professionals. | Mobile Application | 3 months | Baseline, 1-month mid-intervention, 2-months mid-intervention, post-intervention | 1.QoL: SF-36 |
| Pungchompoo et al [29], 2024 | Thailand | Not reported | EG: 24 CG: 30 | Usual care | A home telemedicine model based on website and mobile application containing health education and treatment, the referral system online home visits, and telephone/live counselling. | website + Mobile Application | 6 months | Baseline, post-intervention, 3-months post-intervention, 6-months post-intervention | 1. QoL: The 9‑item Thai Health Status Assessment Instrument |
| Taşkin Duman and Karadakovan [10], 2024 | Turkey | EG: 57.92 ± 13.61 CG: 65.59 ± 10.75 | EG: 26 CG: 22 | Usual care | A 36-sessions video-based training program. | Video | 12 weeks | Baseline, 4-weeks post-intervention, 8-weeks post-intervention, 12-weeks post-intervention | 1.QoL: SF-36 |
| Wang et al [44], 2020 | China | EG: 45.15 ± 7.74 CG: 45.28 ± 7.63 | EG: 74 CG: 74 | Usual care | A WeChat-based teleintervention program, comprising weekly video conferences and fortnightly thematic lectures. | Social media | 12 weeks | Baseline, post-intervention | 1.QoL: SF-36 2.Treatment adherence: Treatment adherence scale for maintenance hemodialysis patients with end-stage renal disease |
| Wang [48], 2021 | China | EG: 60.38 ± 12.01 CG: 64.44 ± 11.62 | EG: 56 CG: 56 | Usual care | A remote intervention based on mindfulness was conducted on WeChat and comprised activities such as meditation, meditating, listening to music. | Social media | 4 weeks | Baseline, post-intervention | 1.Treatment adherence scale for maintenance hemodialysis patients with end-stage renal disease 2.Anxiety: BAI 3.Depression: BDI-II |
| Xi et al [46], 2019 | China | EG: 25.03 ± 4.31 CG: 24.78 ± 4.29 | EG: 20 CG: 20 | Usual care | Making videos on hemodialysis-related health knowledge and care methods and broadcasting them to patients in a loop through a short video app called Tiktok. | Mobile Application | 6 months | Baseline, 3-months mid-intervention, post-intervention | 1.Anxiety: SAS 2.Depression: SDS |
| Zhao et al [45], 2023 | China | EG: 52.49 ± 1.51 CG: 51.38 ± 1.65 | EG: 40 CG: 40 | Usual care | An Internet-based health intervention programme, including relational and therapeutic communication, information support to participants through WeChat. | Social media | 12 months | Baseline, post-intervention | 1.QoL: SF-36 2.Anxiety: SAS 3.Depression: SDS |
| Zhianfar et al [20], 2020 | Iran | Not reported | EG: 35 CG: 35 | Usual care | Use telephone follow-up for peer support, videos and social media to share health knowledge and communication skills. | Telephone + video + Social media | 3 months | Baseline, post-intervention, 1-month post-intervention, 3-months post-intervention | 1.QoL: WHOQOL-SF 2.Treatment adherence: ESRD-AQ 3.Depression: BDI-SF |

Abbreviations: EG: Exprimental group; CG: Control group; QoL: Quality of Life; ESRD-AQ: End-Stage Renal Disease Adherence Questionnaire; EQ-5D: EuroQol 5-Dimension; SF-36: 36-Item Short Form Health Survey; SF-12: 12-Item Short Form Health Survey; KDQOL–SF: Kidney Disease Quality of Life–Short Form; WHOQOL-BREF: The World Health Organization Quality Of Life-BREF; WHOQOL-SF: World Health Organization Quality of Life-SF; DASS: Depression Anxiety and Stress Scale; SAS: Self-Rating Anxiety Scale; BAI: Beck Anxiety Inventory; SDS: Self-rating Depression Scale; BDI-II: Beck Depression Inventory-II; BDI-SF: Beck Depression Inventory-Short Form.
