# Supplementary material for: Efficacy of eHealth Interventions for Hemodialysis Patients: Systematic Review and Meta-Analysis
Source: J Med Internet Res. 2025 Mar 26;27:e67246. doi: 10.2196/67246 (PMC11988279; doi:10.2196/67246)
Supplement: Multimedia Appendix 4 [file jmir_v27i1e67246_app4.docx]

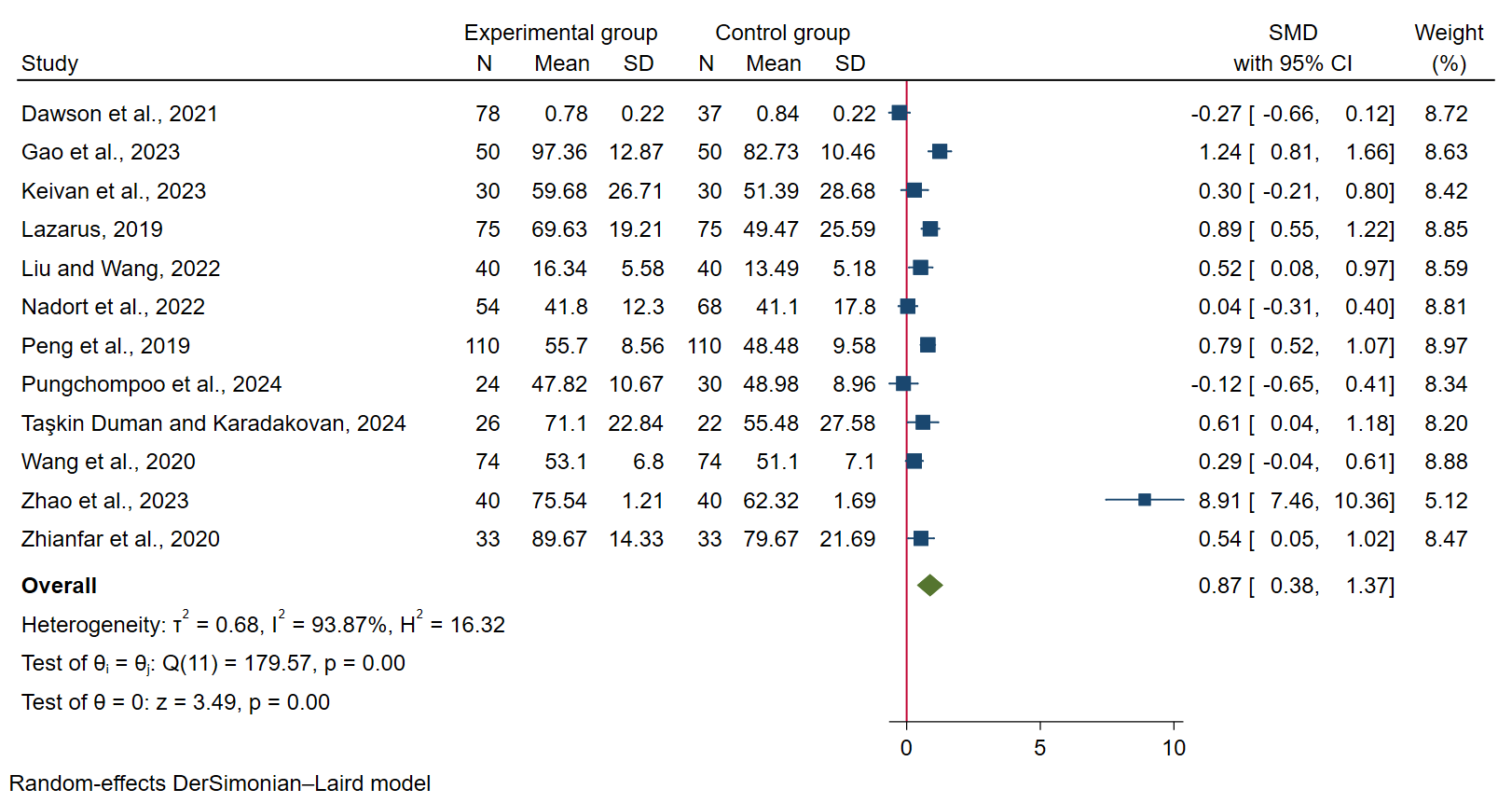


**A-1**


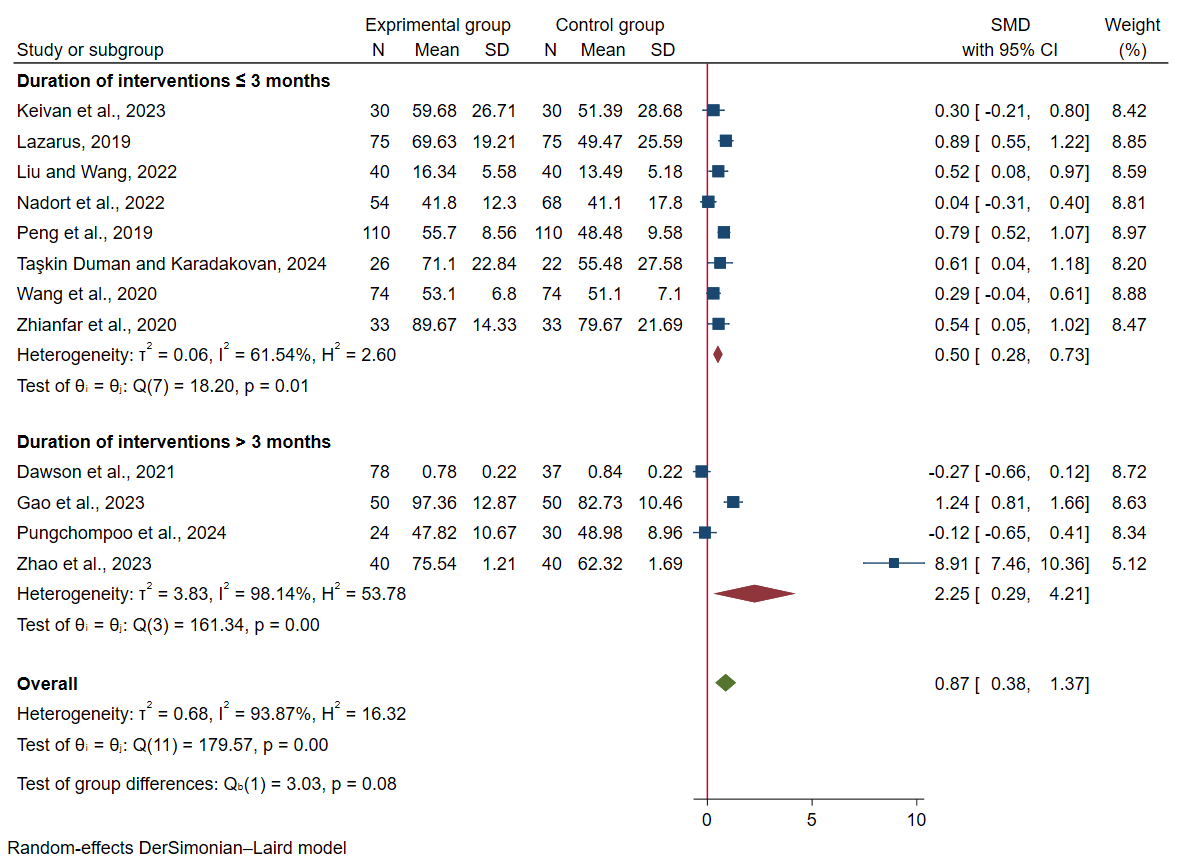


**A-2**


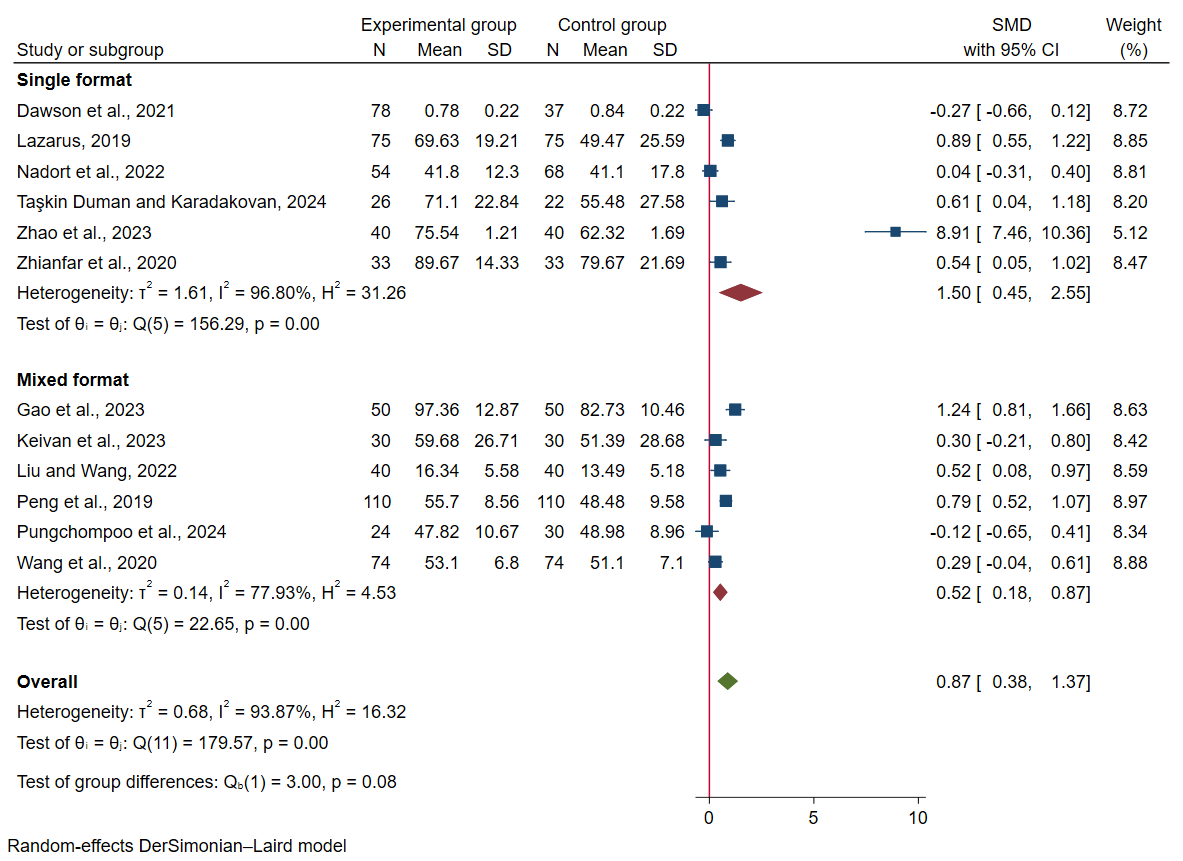


**A-3**


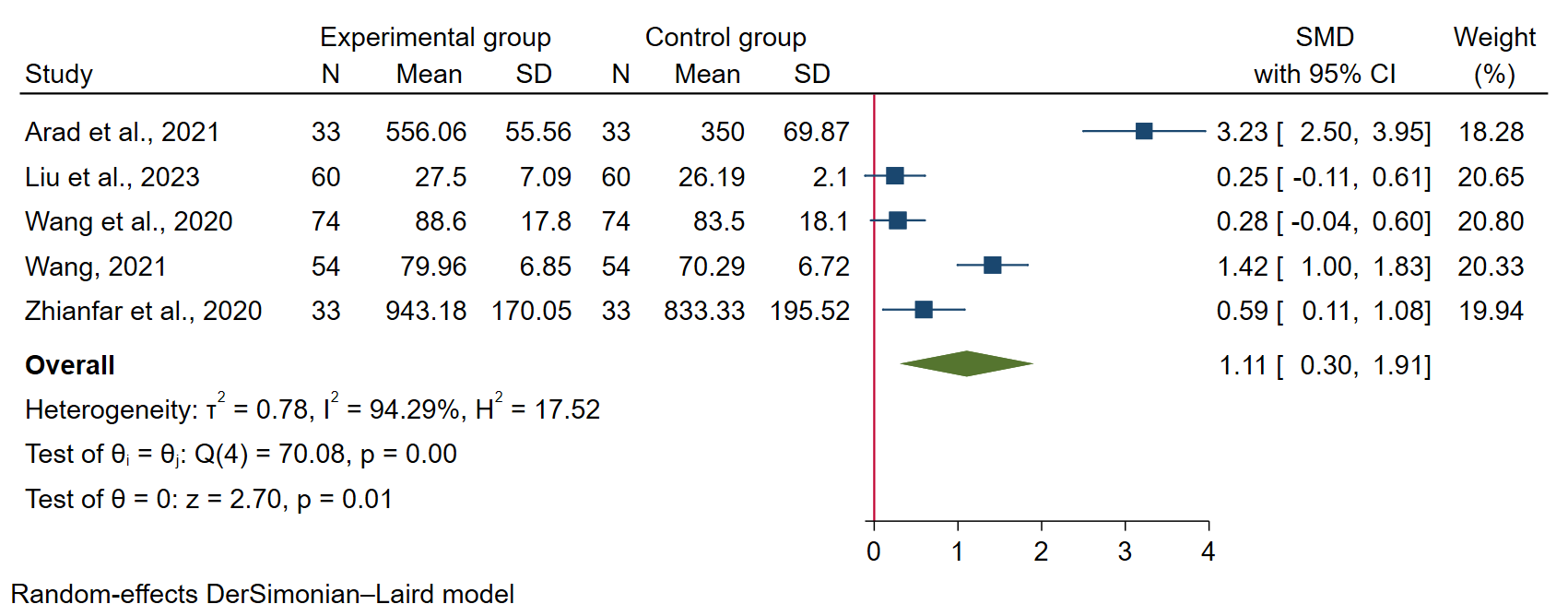


**B-1**


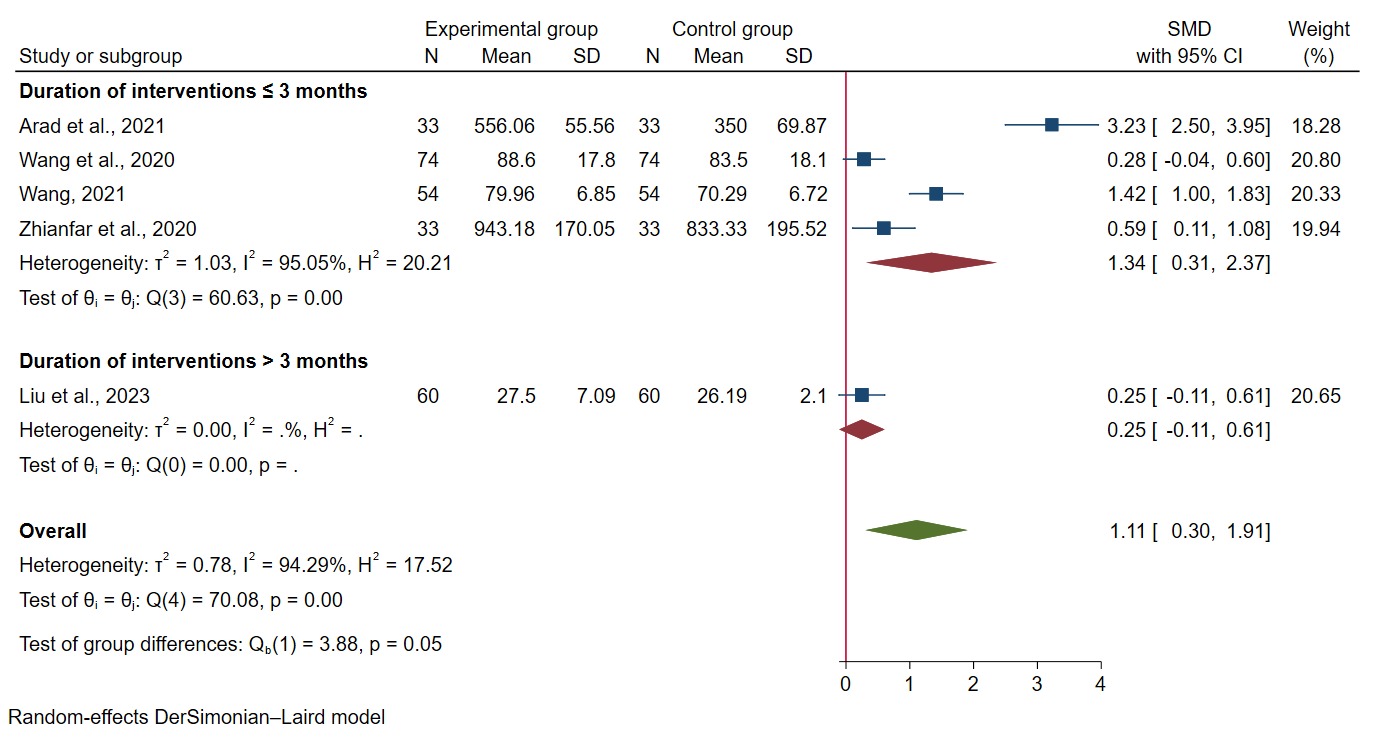


**B-2**


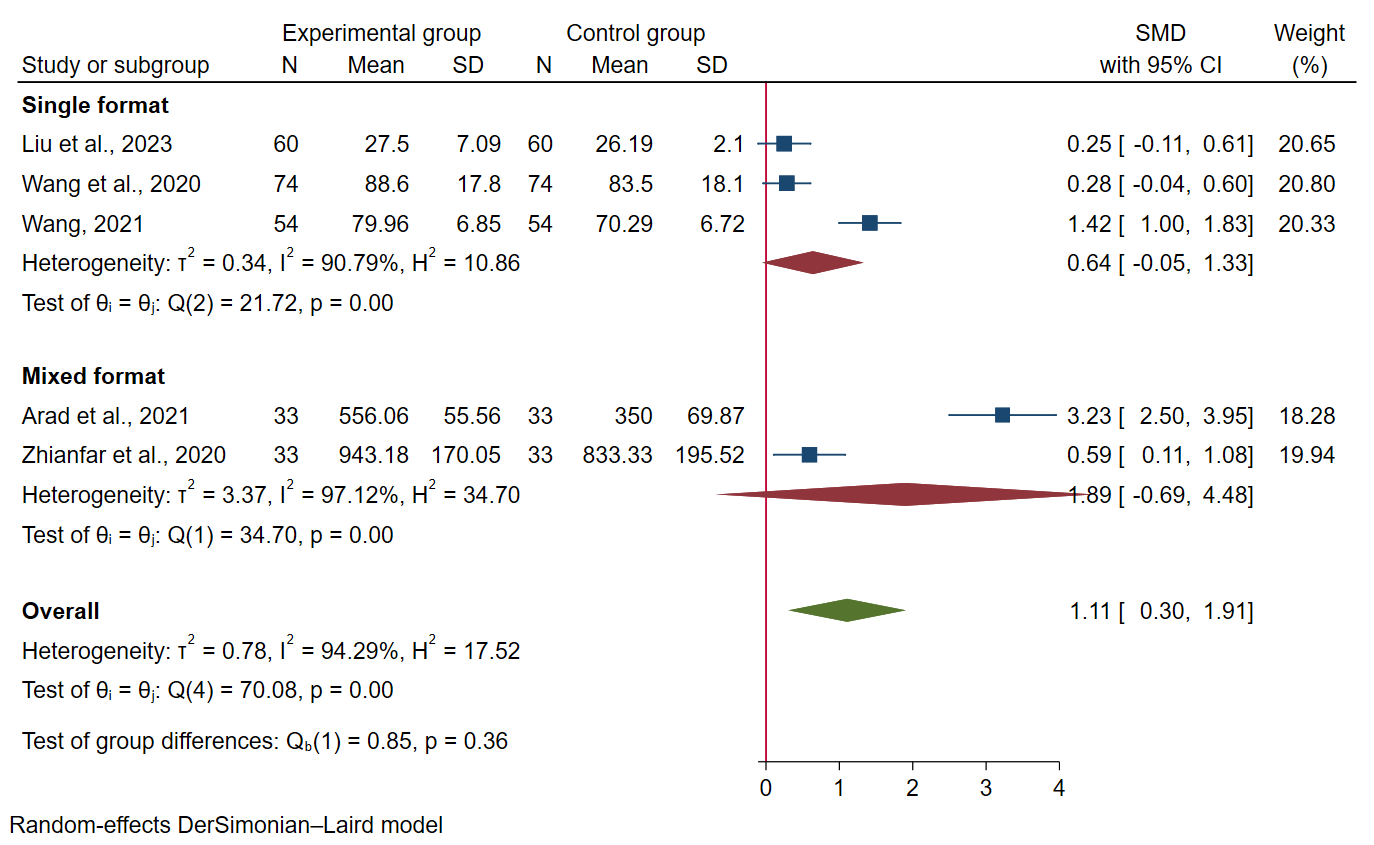


**B-3**


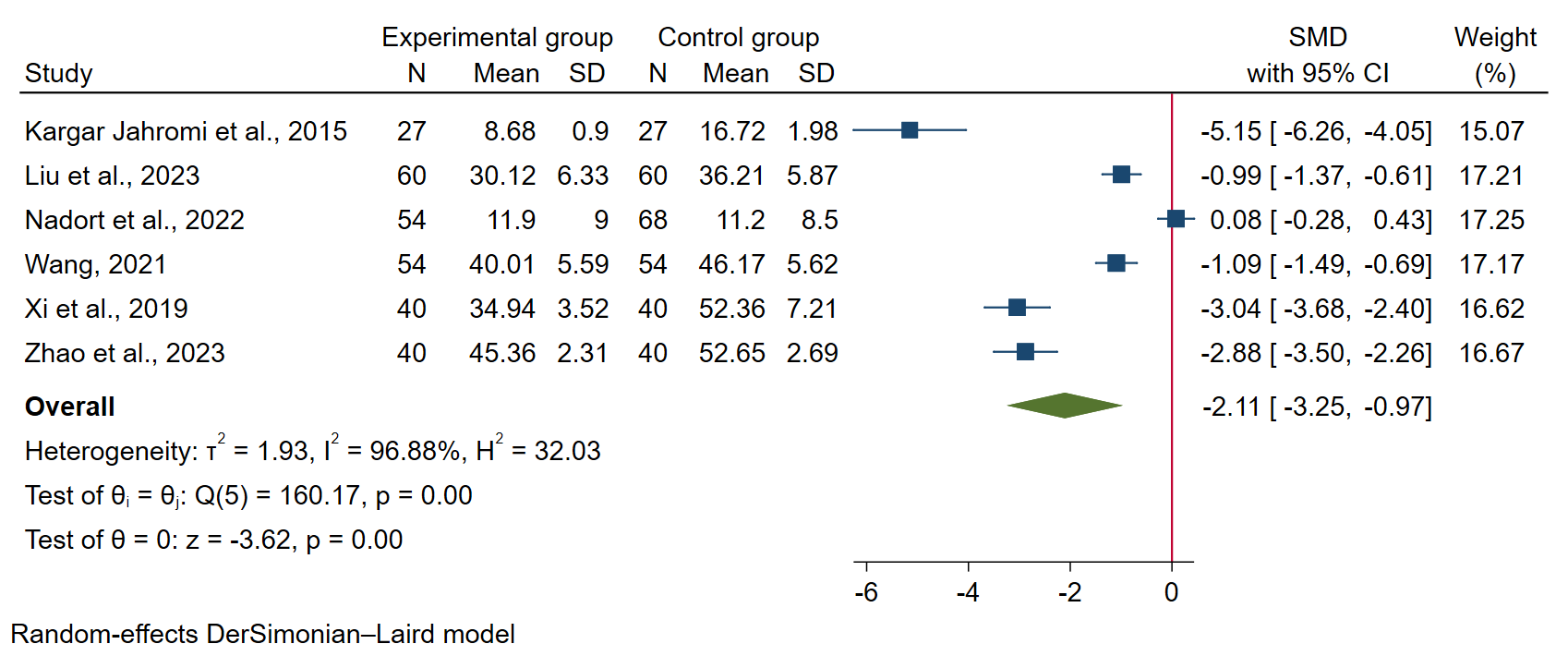


**C-1**


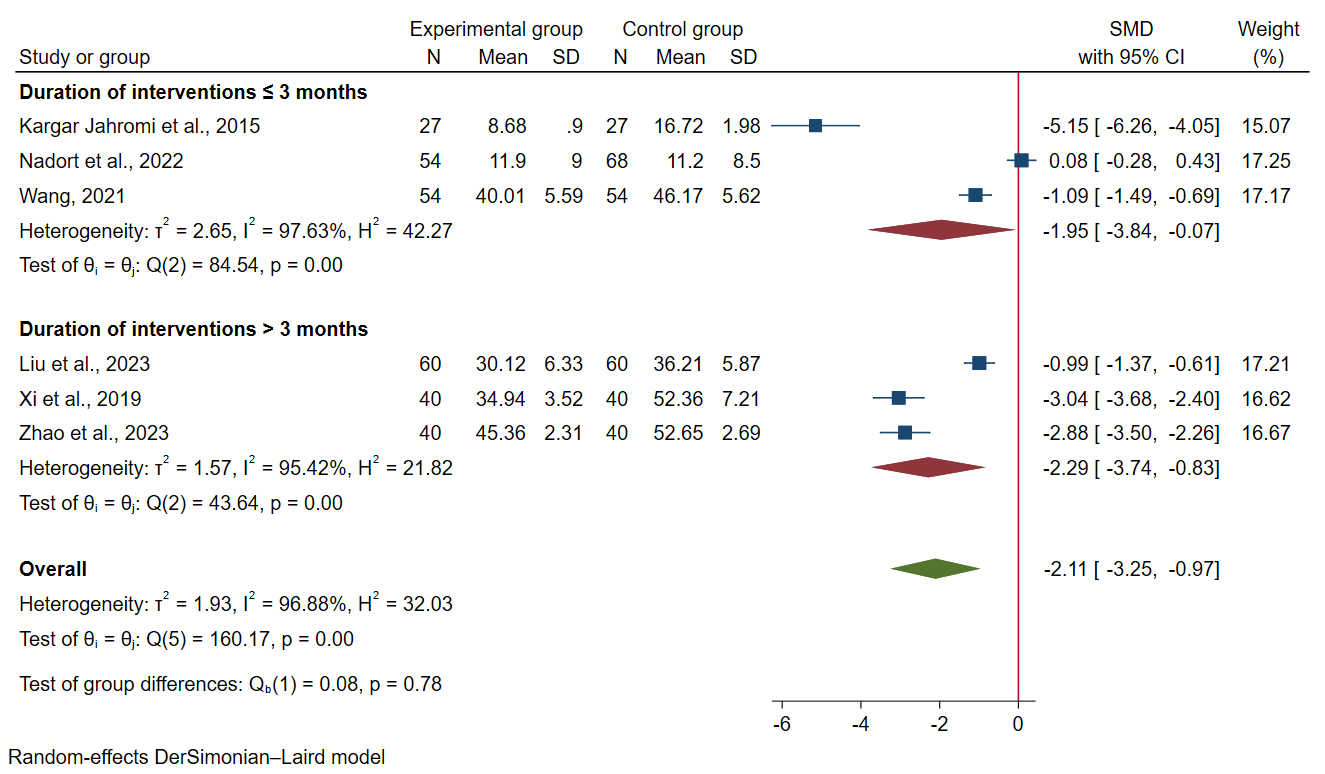


**C-2**


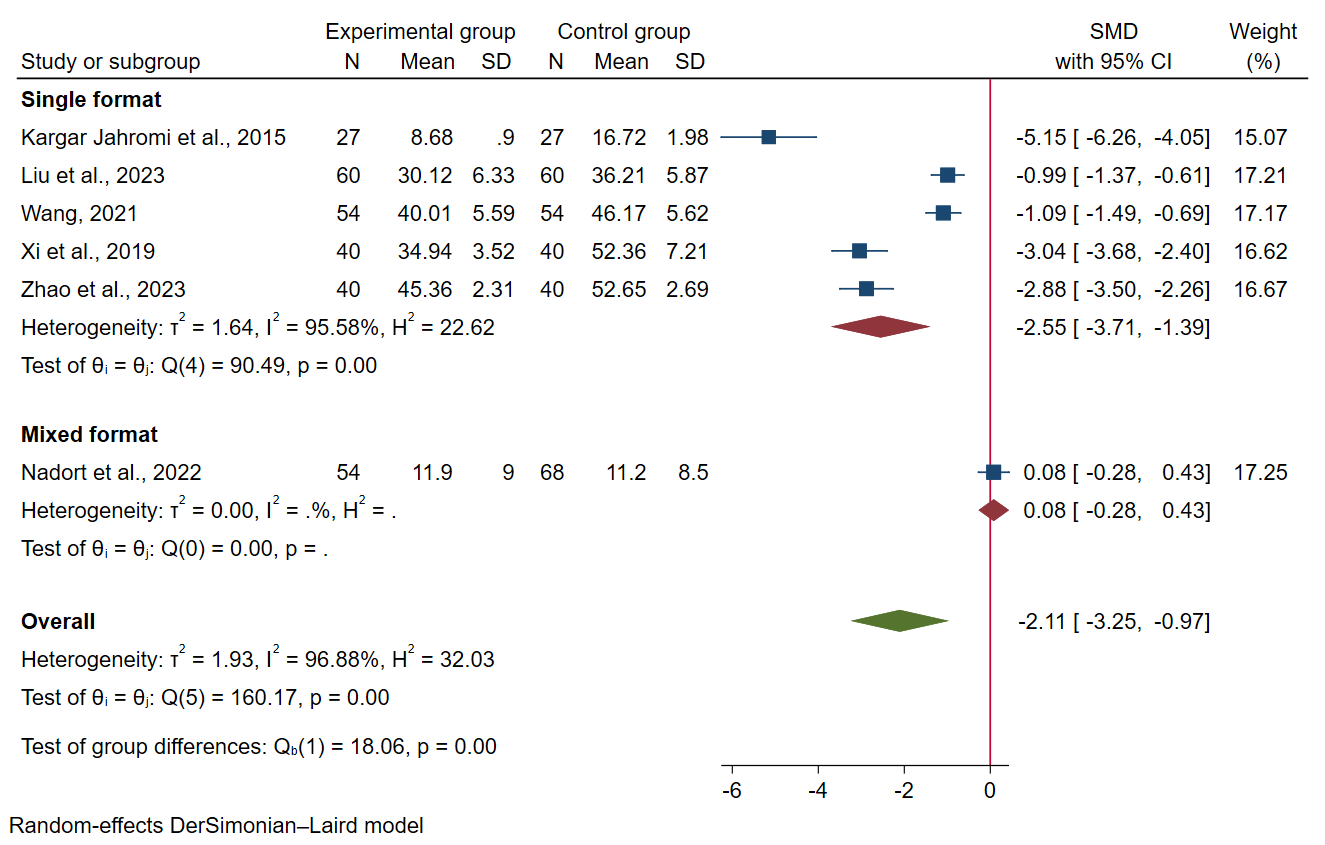


**C-3**


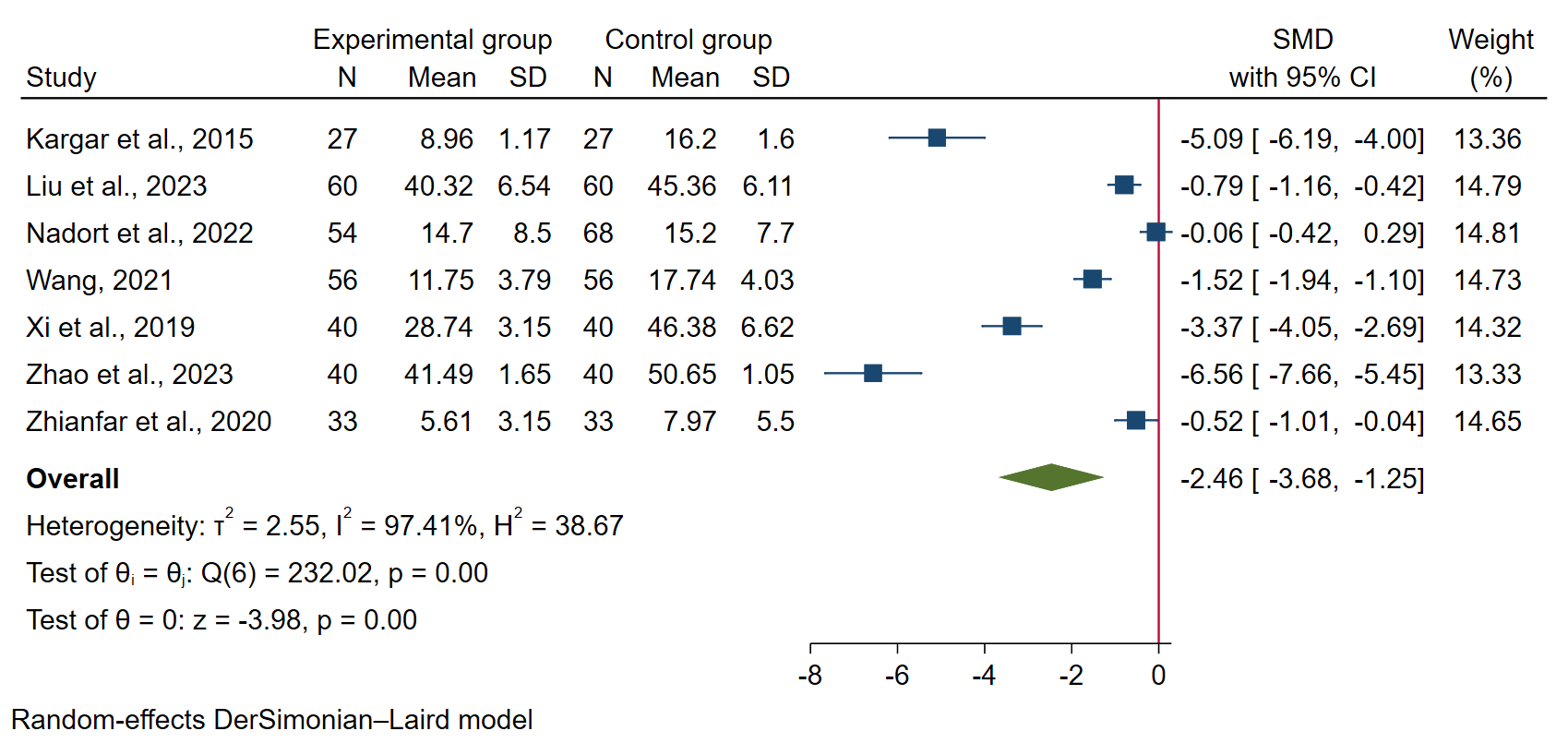


**D-1**


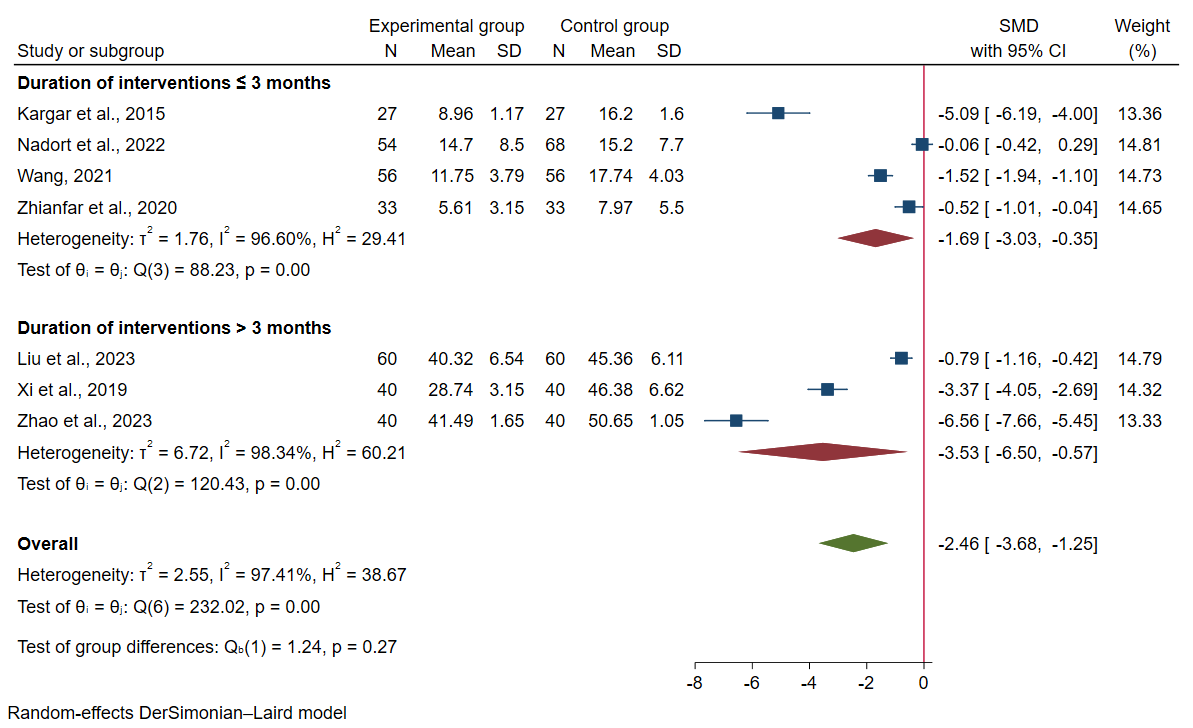


**D-2**


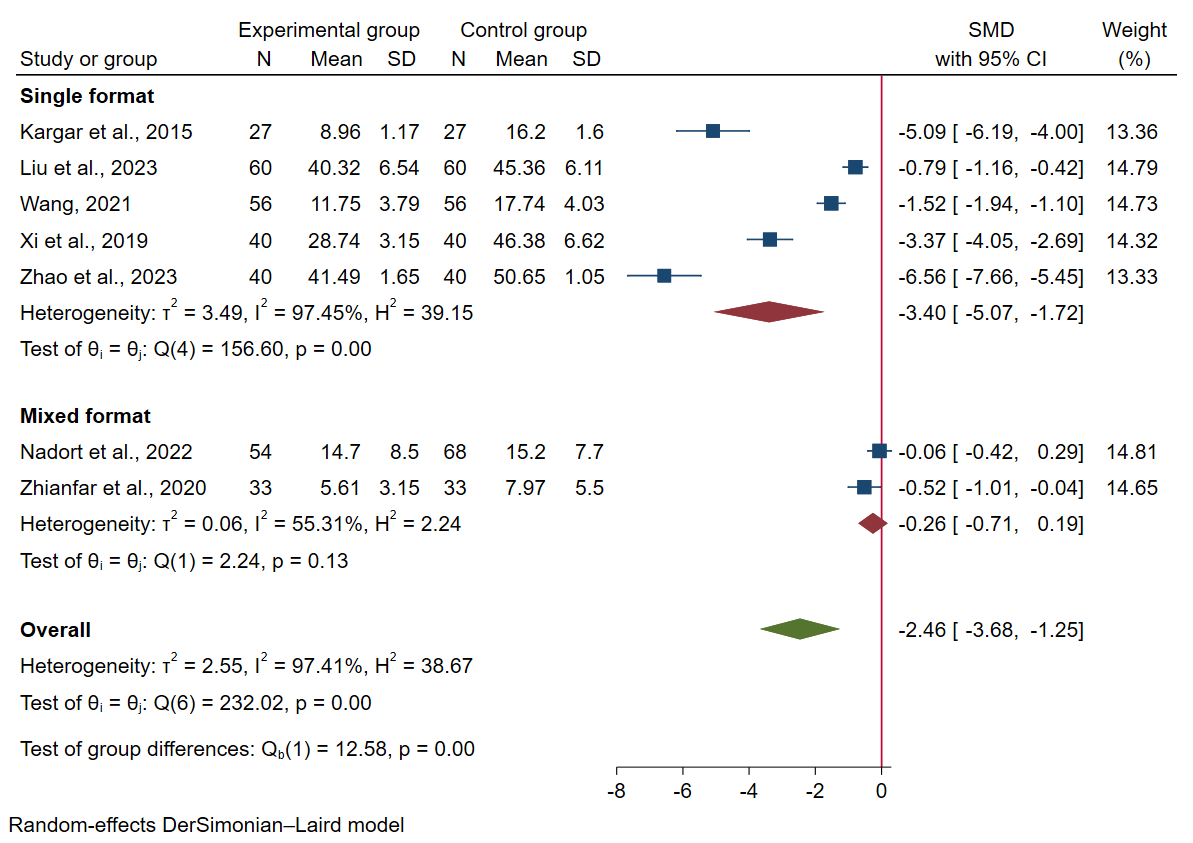


**D-3**

**Figure 3.** Results of meta-analysis: forest plots for the comparison of e-health interventions against usual care.

(A) Quality of life: 1-total effects, 2-grouped by duration of interventions, 3-grouped by formats of interventions;

(B) Treatment adherence: 1-total effects, 2-grouped by duration of interventions, 3-grouped by formats of interventions;

(C) Treatment adherence: 1-total effects, 2-grouped by duration of interventions, 3-grouped by formats of interventions;

(D) Treatment adherence: 1-total effects, 2-grouped by duration of interventions, 3-grouped by formats of interventions;

T²: tau-squared, another measure of heterogeneity;

H²: heterogeneity statistic value, another measure of heterogeneity (related to I² as H² = 1 / (1 - I²);

Q: Q statistic value, used to test for heterogeneity among studies;

θ: a parameter or effect size of interest in the meta-analysis.
