# Supplementary material for: Efficacy of eHealth Interventions for Hemodialysis Patients: Systematic Review and Meta-Analysis
Source: J Med Internet Res. 2025 Mar 26;27:e67246. doi: 10.2196/67246 (PMC11988279; doi:10.2196/67246)
Supplement: Multimedia Appendix 5 [file jmir_v27i1e67246_app5.doc]

**Multimedia Appendix 5. Sensitivity analyses for quality of life, treatment adherence, anxiety, and depression**

**
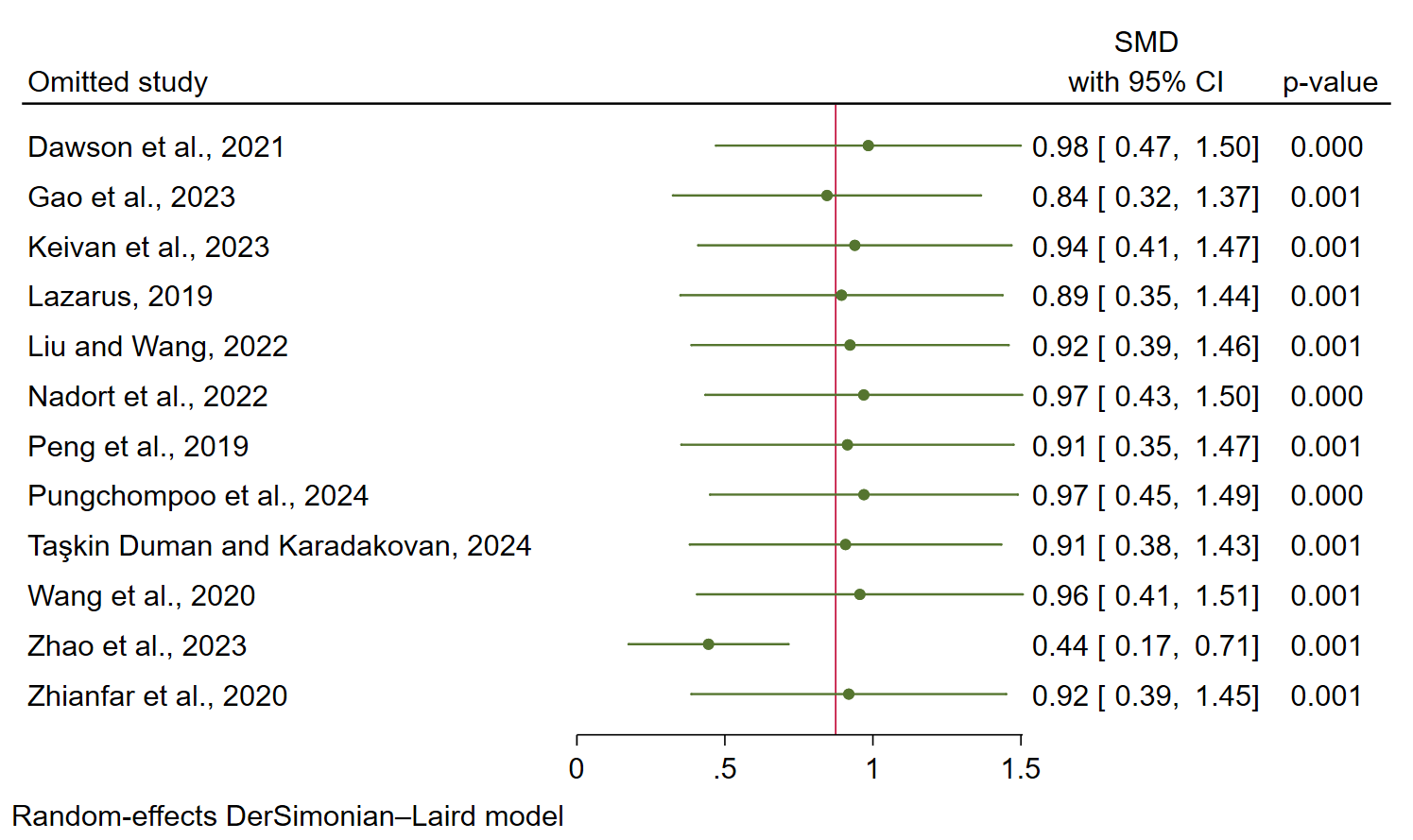
**

Fig. S2a. Sensitivity test for quality of life.

**
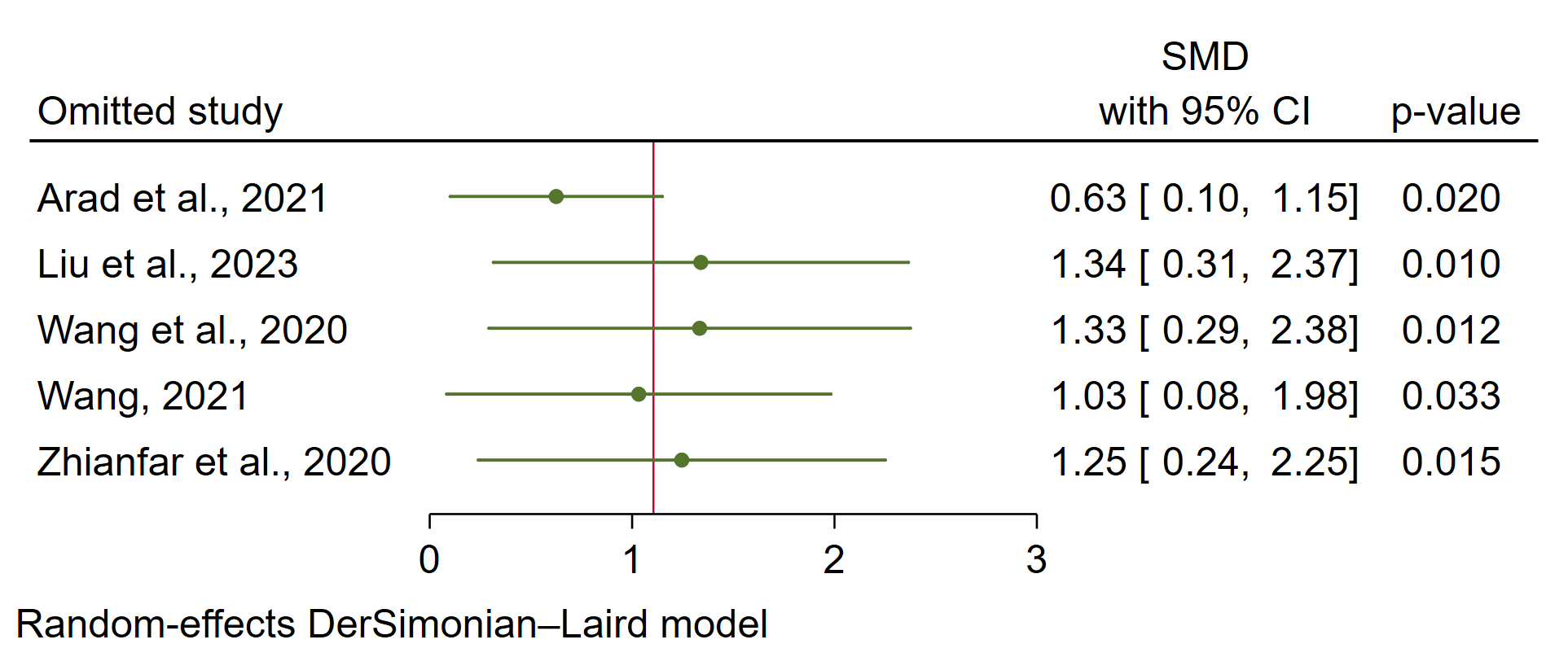
**

Fig. S2b. Sensitivity test for treatment adherence.

**
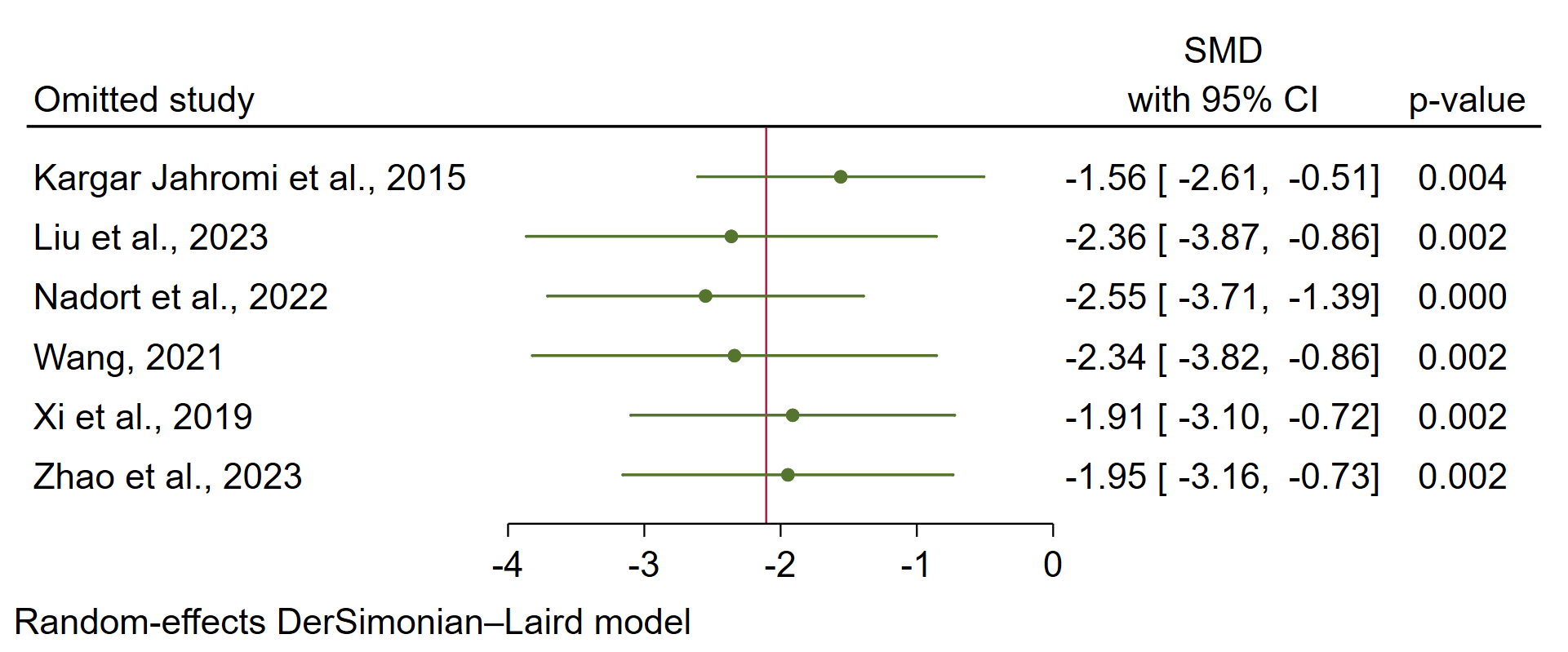
**

Fig. S2c. Sensitivity test for anxiety.

**
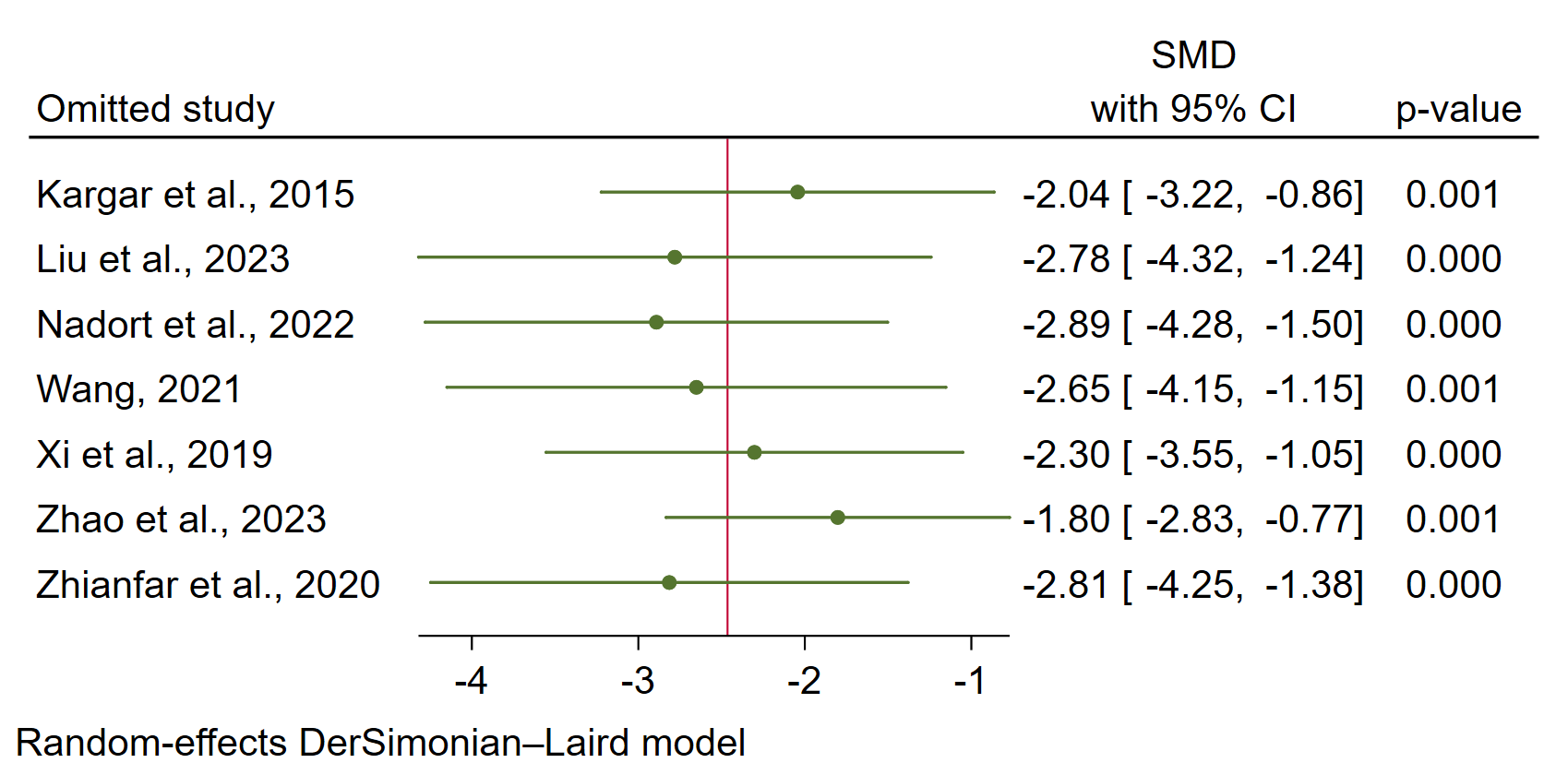
**

Fig. S2d. Sensitivity test for depression.
