# Supplementary figures and images for: Efficacy of eHealth Interventions for Hemodialysis Patients: Systematic Review and Meta-Analysis
Source: J Med Internet Res. 2025 Mar 26;27:e67246. doi: 10.2196/67246 (PMC11988279; doi:10.2196/67246)

**Multimedia Appendix 6. Funnel plot**


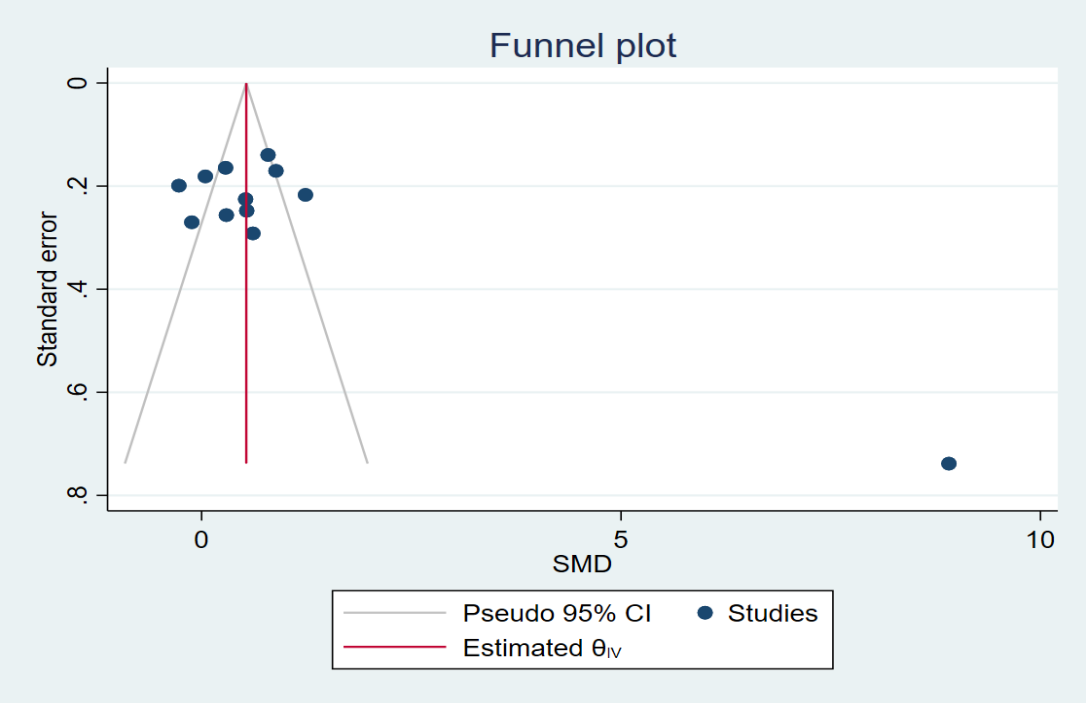


Figure S3. Funnel plot

Supplement: Multimedia Appendix 6 [file jmir_v27i1e67246_app6.docx]
